# In vitro CB_1_ receptor activity of halogenated indazole synthetic cannabinoid receptor agonists

**DOI:** 10.1007/s00204-025-04082-4

**Published:** 2025-05-18

**Authors:** Henrik Green, Craig McKenzie, Elias Hamra, Tobias Rautio, Xiongyu Wu, Emma Juneskog, Rebecka Sandblom, Manuela Carla Monti, Mattias Persson, Caitlyn Norman

**Affiliations:** 1 Department of Biomedical and Clinical Science, Division of Clinical Chemistry and Pharmacology, Linköping University, Linköping, Sweden; 2 Department of Forensic Genetics and Forensic Toxicology, National Board of Forensic Medicine, Linköping, Sweden; 3 Chiron AS, Trondheim, Norway; 4 Department of Physics, Chemistry and Biology, Linköping University, Linköping, Sweden

**Keywords:** Halogenated synthetic cannabinoid receptor agonists, New psychoactive substances, CB_1_ cannabinoid receptor, Potency, Structure–activity relationships

## Abstract

Synthetic cannabinoid receptor agonists (SCRAs) are a group of new psychoactive substances (NPS) that bind to and activate the cannabinoid 1 and 2 (CB_1_ and CB_2_) receptors. Following the introduction of SCRA analog controls in China in July 2021, new SCRAs with a bromine at the 5 position on the indazole core emerged on the recreational drug market. In this study, the in vitro CB_1_ receptor activity of 19 different SCRAs with a halogenated indazole core was systematically examined using a AequoScreen^®^ CB_1_ assay alongside four available non-halogenated analogs, ADB-BUTINACA, MDMB-BUTINACA, MDMB-4en-PINACA, and MDMB-INACA, and the newly emerged MDMB-5′Me-INACA (MDMB-5-methyl-INACA). Of SCRAs with a substitution at the 5 position on the indazole core, analogs with a fluorine had the lowest EC_50_ values. For SCRAs with a methyl ester head moiety (MMB or MDMB), chlorinated analogs had the next lowest EC_50_ values followed by brominated analogs; however, the opposite relationship was observed for SCRAs with an amide head moiety (AB or ADB). All halogenated compounds had similar potency to their non-halogenated analog, except the brominated *tert*-leucine methyl ester SCRAs, which had significantly reduced potency. Of the different head moieties, *tert*-leucine methyl ester SCRAs were the most potent, followed by *tert*-leucinamide, valinamide, and valine methyl ester. The potencies of the SCRAs with pentyl (ADB-5′Br-PINACA/ADMB-5′Br-PINACA/ADB-P-5′Br-INACA), pent-4-enyl (ADB-4en-5′Br-PINACA/ADMB-4en-5′Br-PINACA/ADB-4en-P-5′Br-INACA and MDMB-4en-5′Br-PINACA/MDMB-4en-P-5′Br-INACA), and butyl tails (ADB-5′Br-BUTINACA/ADMB-5′Br-BUTINACA/ADB-B-5′Br-INACA) were not significantly different, whereas fluorobenzyl (ADB-5′Br-FUBINACA), tail-less (ADB-5′Br-INACA), and decyl tail (ADB-5′Br-DECINACA/ADMB-5′Br-DECINACA/ADB-D-5′Br-INACA) analogs were significantly less potent.

**Supplementary Information:**

The online version contains supplementary material available at 10.1007/s00204-025-04082-4.

## Introduction

Synthetic cannabinoid receptor agonists (SCRAs) are a large group of new psychoactive substances (NPS) that bind to and activate the cannabinoid 1 and 2 (CB_1_ and CB_2_) receptors, which are Class A G protein-coupled receptors (GPCRs) (Matsuda et al. 1990; Munro et al. 1993; Pertwee 2006). SCRAs are designed to mimic the effects of Δ^9^-tetrahydrocannabinol (THC), the main psychoactive component of cannabis; however, they are often much more potent and can have adverse effects more closely related to psychostimulants than cannabis, including psychosis, aggression, addiction, acute kidney injury, and even death (Darke et al. 2021; Giorgetti et al. 2020; Manini et al. 2022).

As they are designed to be legal alternatives to cannabis, the SCRAs available on the illicit market are continuously evolving in response to national and international legislation, particularly in producer countries such as China (Norman et al. 2021). SCRAs emerging on the illicit market are typically closely related analogs to those controlled, but with alteration(s) to at least one of the four main structural components: the tail, core, linker, or head (linked group). The tail and head moieties are the most commonly altered while the linker and the core tend to be the most consistent structural components (Pulver et al. 2022). However, in July 2021, analog controls on the most commonly observed SCRA structural scaffolds were introduced in China. This led to the emergence of SCRAs with a new acetamide linker (e.g., CH-PIATA) (Deventer et al. 2022a; Norman et al. 2023), as well as new core moieties, including the monocyclic pyrazole (e.g., 5 F-3,5-AB-PFUPPYCA) (Deventer et al. 2023a, b), oxoindolin (e.g., BZO-HEXOXIZID) (Deventer et al. 2022b), and brominated indazole (e.g., ADB-5′Br-BUTINACA) (Choi et al. 2022; Marland et al. 2024).

Analogs of indazole-3-carboxamide SCRAs with a bromine on the indazole core were included in a 2009 Pfizer patent (Buchler et al. 2009), but such compounds were not detected on the recreational drug market until 2021. Two brominated SCRAs without a tail moiety, ADB-5′Br-INACA (*N*-[1-amino-3,3-dimethyl-1-oxobutan-2-yl]−5-bromo-1*H*-indazole-3-carboxamide; also known as ADMB-5′Br-INACA) (European Monitoring Centre for Drugs and Drug Addiction (EMCDDA) 2022a), MDMB-5′Br-INACA (methyl 2-[5-bromo-1*H*-indazole-3-carboxamido]−3,3-dimethylbutanoate) (EMCDDA, 2022b), and ADB-5′Br-BUTINACA (*N*-[1-amino-3,3-dimethyl-1-oxobutan-2-yl]−5-bromo-1-butyl-1*H*-indazole-3-carboxamide; also known as ADB-B-5′Br-INACA, ADB-5′Br-BINACA, and ADMB-5′Br-BUTINACA) (EMCDDA 2022c) were first reported to the EU Early Warning System in 2022. These brominated SCRA analogs have also been detected outside of the EU in the US (de Campos et al. 2024; Norman et al. 2024), Scotland (Norman et al. 2024), New Zealand (Knowyourstuffnz 2022), South Korea (Choi et al. 2022; Kim et al. 2023), and Kuwait (Al-Matrouk and Orabi 2024).

Since these first detections, brominated indazole SCRAs have become increasingly prevalent with four more analogs reported to the EU Early Warning System: ADB-5′Br-DECINACA (*N*-(1-amino-3,3-dimethyl-1- oxobutan-2-yl)−5-bromo-1-decyl-1*H*-indazole-3-carboxamide; also known as ADMB-5′Br-DECINACA and ADB-D-5′Br-INACA) (EMCDDA 2022 d) in 2022, ADB-5′Br-PINACA (*N*-(1-amino-3,3-dimethyl-1-oxobutan-2-yl)−5-bromo-1-pentyl-1*H*-indazole-3-carboxamide; also known as ADMB-5′Br-PINACA and ADB-P-5′Br-INACA) (EMCDDA) 2022e) in 2022, ADB-4en-5′Br-PINACA (*N*-(1-amino-3,3-dimethyl-1-oxobutan-2-yl)−5-bromo-1-(pent-4-en-1-yl)−1*H*-indazole-3-carboxamide; also known as ADMB-4en-5′Br-PINACA and ADB-4en-P-5′Br-INACA) (EMCDDA) 2022f) in 2022, and MDMB-4en-5′Br-PINACA (methyl 2-{[5-bromo-1-(pent-4-en-1-yl)−1*H*-indazole-3-carbonyl]amino}−3,3-dimethylbutanoate; also known as MDMB-4en-P-5′Br-INACA) in November 2024 (European Union Drugs Agency (EUDA) 2024). In addition, a SCRA with a methyl group at the 5 position on the indazole core, MDMB-5′Me-INACA (methyl 3,3-dimethyl-2-[(5-methyl-1*H*-indazole-3-carbonyl)amino]butanoate; also known as MDMB-5-methyl-INACA), was detected in seized drug material in the US in August 2023 (Krotulski et al. 2023).

While halogenation is a common modification used during pharmaceutical drug development to enhance the potency of drugs, for SCRAs emerging onto the illicit market before 2021, halogenation, most commonly fluorination, had largely been confined to substitution on the tail moiety. Terminal fluorination of the tail moiety of SCRA analogs has been one of the most commonly observed structural alterations and has been found to increase the in vitro potency about 2–5 times at the CB_1_ receptor, the receptor responsible for the majority of the psychoactive effects of SCRAs (Banister et al. 2015). In comparison, there is little information available on the effect of the halogenation or substitution of the indazole core at the 5 position on the in vitro CB_1_ receptor potency. Using an AequoScreen^®^ assay, Deventer et al. (2023b) found the in vitro CB_1_ receptor potency of ADB-5′Br-BUTINACA (EC_50_ = 12.5 nM) was similar to its non-brominated analog (ADB-BUTINACA EC_50_ = 11.5 nM) and the potency of the fluorinated analog was slightly reduced (ADB-5′F-BUTINACA EC_50_ = 18.3 nM). However, a CB_1_ β-arrestin 2 recruitment assay found opposing results, where the fluorinated analog (ADB-5′F-BUTINACA EC_50_ = 28.8 nM), was more potent than the brominated analog (ADB-5′Br-BUTINACA EC_50_ = 82.1 nM) (Deventer et al. 2023b), although both were still less potent than the non-halogenated analog (ADB-BUTINACA EC_50_ = 7.72 nM) (Sparkes et al. 2022). This demonstrates some inconsistencies in structure–activity relationships (SARs) of the halogenation of the indazole core that need to be explored further, particularly given the increasing emergence of new indazole-3-carboxamide SCRAs with a substitution at the 5 position on the indazole core.

**Fig. 1:**
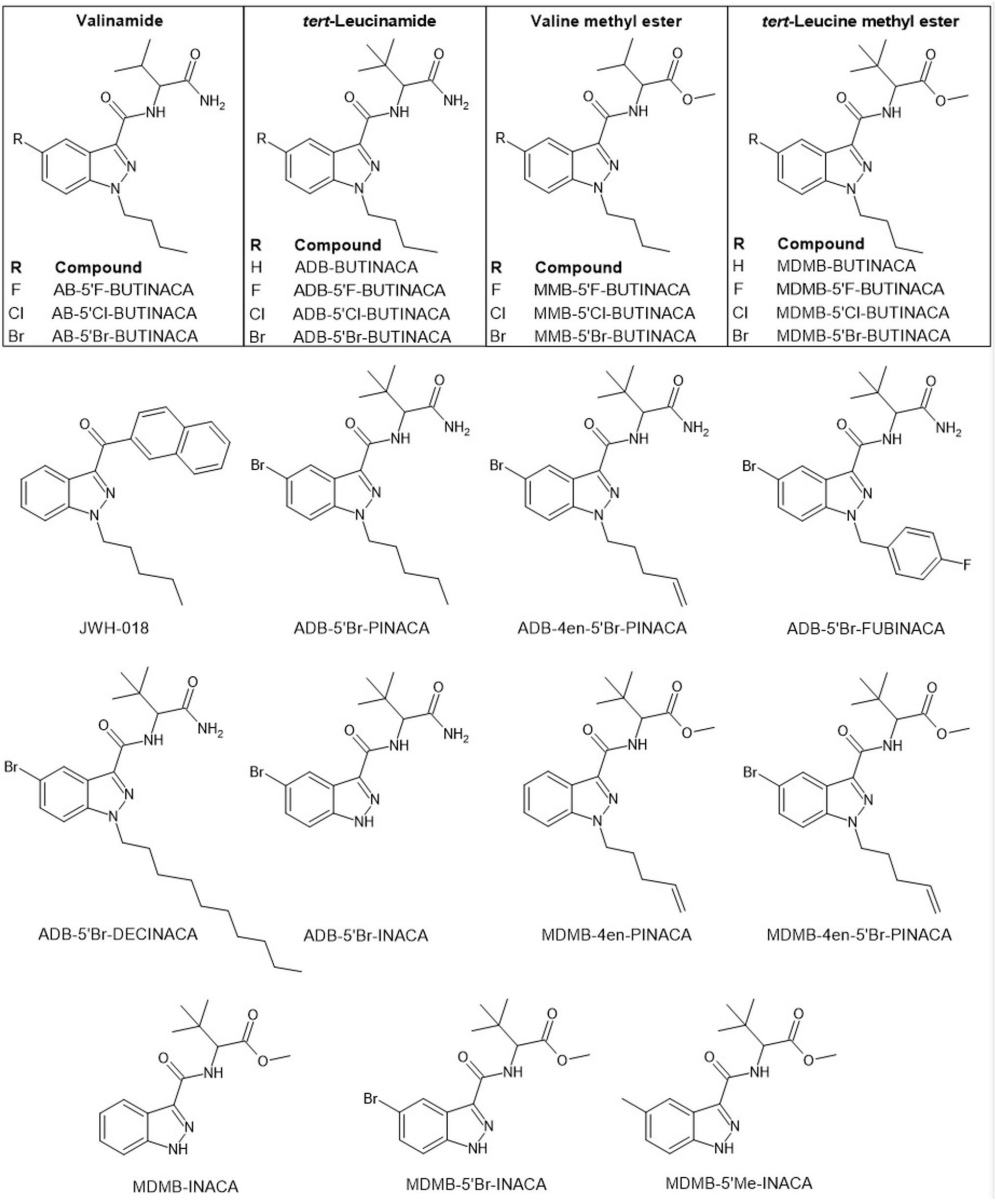
Structures of JWH-018 and the 24 synthetic cannabinoid receptor agonists (SCRAs) examined in this study. All reference standards used were racemic mixtures, so the structures are presented without their chirality

In this study, the in vitro CB_1_ receptor activity of 24 different SCRAs, including 19 SCRAs with a halogenated indazole core (see structures in Fig. 1) and their four available non-halogenated analogs, ADB-BUTINACA, MDMB-BUTINACA, MDMB-4en-PINACA, and MDMB-INACA, was systematically examined using an AequoScreen^®^ CB_1_ assay. In addition, the in vitro CB_1_ receptor activity of the newly emerged MDMB-5′Me-INACA was examined. The SARs of the different substitutions on the indazole core, as well as head and tail moieties were explored.

## Materials and methods

### Chemicals and reagents

The AequoScreen^®^ recombinant Chinese hamster ovary (CHO) K1 cell line, stably expressing the human CB_1_ receptor (ES-110-A), was obtained from Revvity (Sollentuna, Sweden). DMEM/Ham’s F12, Ham’s F12, 0.25% trypsin–EDTA with phenol red, and fetal bovine serum (FBS) were purchased from Thermo Fisher (Gothenburg, Sweden). HEPES buffer, l-glutamine, protease-free bovine serum albumin (BSA), digitonin, adenosine-5′-triphosphate disodium salt hydrate (ATP), methanol, and DMSO were procured from Sigma-Aldrich (Stockholm, Sweden). The coelenterazine substrate was from Nanolight Technology (Pinetop, AZ, United States). JWH-018 was obtained from Chiron AS (Trondheim, Norway). MDMB-BUTINACA, MDMB-INACA, MDMB-4en-5′Br-PINACA, MDMB-5′Me-INACA, and ADB-5′Br-DECINACA reference standards (purity ≥ 98%) were purchased from Cayman Chemical (Ann Arbor, MI, USA). All reference standards were racemic mixtures.

### Synthesis of SCRA reference standard

Reference standards for AB-5′Br-BUTINACA, AB-5′F-BUTINACA, AB-5′Cl-BUTINACA, MMB-5′Br-BUTINACA, MMB-5′F-BUTINACA, MMB-5′Cl-BUTINACA, ADB-5′Br-BUTINACA, ADB-5′F-BUTINACA, ADB-5′Cl-BUTINACA, MDMB-5′Br-BUTINACA, MDMB-5′F-BUTINACA, MDMB-5′Cl-BUTINACA, ADB-5′Br-PINACA, ADB-4en-5′Br-PINACA, ADB-5′Br-FUBINACA, and ADB-5′Br-INACA were synthesized in-house as described previously (Rautio et al. 2024). Details of the synthesis and characterization data can be found in the Supplementary Information (Figures S1, S2). All reference standards were synthesized as racemic mixtures.

### In vitro biological activity at CB_1_ receptor

The apoaequorin system (AequoScreen^®^ assay) is an intracellular calcium (Ca^2+^) release assay commonly used for measuring GPCR activity. Apoaequorin is a protein that in the presence of its substrate coelenterazine converts to the aequorin photoprotein, which has three Ca^2+^-binding sites (Bakayan et al. 2016; Deng et al. 2009). Binding of an extracellular ligand to the GPCR leads to the activation of a universal G protein subunit G_α16_, which in turn triggers a series of downstream events, such as the activation of the phospholipase C (PLC) enzyme, that stimulates the release of intracellular Ca^2+^ (Deng et al. 2009; Shimomura et al. 1974; Stables et al. 1997). The Ca^2+^ then binds to the aequorin photoprotein, which oxidizes the coelenterazine leading to the release of photons that can be measured via a luminescence reader (Wouters et al. 2019).

CHO-K1 cells stably expressing CB_1_, the apoaequorin enzyme, and the G_α16_ subunit were maintained in a humidified atmosphere at 37˚C and 5% CO_2_ in Ham's F12 medium supplemented with 10% heat-inactivated FBS. To perform the assays, cells were trypsinized (10 min, 37˚C), centrifuged (at 200 × *g*, 5 min, room temperature), counted, and resuspended at 3 × 10^5^ cells/mL in DMEM/Ham's F12 without phenol red, and supplemented with 15 mM HEPES, L-glutamine, and protease-free BSA (0.1%) (further referred to as assay medium). The coelenterazine substrate was added to a final concentration of 2.5 μM and the suspension was incubated for 3 h (room temperature, rotating at ~ 7 RPM/min, protected from light). Drug solutions were prepared as a 1:8 serial dilution in assay medium with a starting concentration of 60 μM (in well after addition of cells) and then added to white, opaque-welled 96-well plates. JWH-018 (60 μM) was included as a reference on each plate, in line with earlier results generated using the AequoScreen^®^ CB_1_ assay (Deventer et al. 2023b; Kronstrand et al. 2022; Truver et al. 2020). Digitonin (67 μM) and ATP (6.7 μM) were included on each plate and served as positive controls for coelenterazine loading as both are involved in the non-CB-dependent release of calcium ions. Blank assay medium was used as a negative control. Using a TECAN Spark 10 M plate reader (Männedorf, Switzerland), 50 μL of the incubated cell suspension was dispensed into each well (15 × 10^3^ cells/well) of the 96-well plate containing the test solutions. Luminescence was measured for 25 s (corresponding to 190 additional reading cycles).

### Data analysis

Absolute luminescence signals were corrected for intra-plate variability in Microsoft Excel 365 using area under the curve (AUC) values of JWH-018 and calculated for each concentration of the test compounds. Values were then blank-corrected by subtracting AUC values of the mean of the blank controls. Data were normalized to the maximum of JWH-018. Normalized values were transferred to GraphPad Prism (Version 10.0.2) to generate concentration–response curves and calculate EC_50_ and *E*_max_ values by curve fitting via nonlinear regression (three-parameter logistic fit). Results are represented as receptor activity of JWH-018 (%) derived from a minimum of three independent experiments (*n* ≥ 3), run in triplicate. Brown–Forsythe and Welch ANOVA tests (*α* = 0.05) were performed in GraphPad Prism to compare the EC_50_ and *E*_max_ values between all compounds.

## Results and discussion

### In vitro activity

The relative efficacy (*E*_max_) and the potency (EC_50_) values calculated for the 24 SCRAs examined in this study and the reference compound JWH-018 are provided in Table 1. The p values reported in Table 1 are from a Brown–Forsythe and Welch ANOVA tests (*α* = 0.05) comparing each compound to the reference compound JWH-018. Brown–Forsythe and Welch ANOVA tests (*α* = 0.05) were also performed comparing each compound to all of the other compounds. The results of these tests are discussed below and the full data can be found in the Supplementary Information (Section S3).

**Table 1:** Relative efficacy (*E*_max_) and potency (EC_50_) calculated for the 24 SCRAs examined in this study and reference compound (JWH-018). *P* values are from Brown-Forsythe and Welch ANOVA tests (*α* = 0.05) with JWH-018 as a reference

Compound	Structural components			Efficacy (% of JWH-018)			Potency (EC_50_, nM)		
	Head	Core substitution	Tail	*E*_max_	95% CI	*P* value	EC_50_	95% CI	*P* value
JWH-018	–	–	–	99.7	97.7–102	–	20.6	17.8–23.9	–
AB-5′F-BUTINACA	AB	F	Butyl	107	105–110	0.10	18.7	15.7–22.2	1.00
AB-5′Cl-BUTINACA	AB	Cl	Butyl	112	109–116	0.07	57.7	47.7–69.9	0.02
AB-5′Br-BUTINACA	AB	Br	Butyl	115	112–118	0.04	44.6	37.0–53.6	0.04
ADB-BUTINACA	ADB	–	Butyl	126	123–129	< 0.01	21.5	18.0–25.6	> 1.00
ADB-5′F-BUTINACA	ADB	F	Butyl	113	109–117	0.08	14.4	11.1–18.8	0.60
ADB-5′Cl-BUTINACA	ADB	Cl	Butyl	113	109–116	0.05	30.1	24.8–36.6	0.40
ADB-5′Br-BUTINACA	ADB	Br	Butyl	110	108–112	0.03	18.0	15.5–21.0	1.00
MMB-5′F-BUTINACA	MMB	F	Butyl	107	105–109	0.08	26.4	23.1–30.3	0.50
MMB-5′Cl-BUTINACA	MMB	Cl	Butyl	109	106–113	0.10	148	124–176	< 0.01
MMB-5′Br-BUTINACA	MMB	Br	Butyl	105	100–111	0.71	259	195–346	< 0.01
MDMB-BUTINACA	MDMB	–	Butyl	121	118–125	0.01	8.90	7.27–10.9	< 0.01
MDMB-5′F-BUTINACA	MDMB	F	Butyl	111	109–114	0.02	5.75	4.86–6.81	< 0.01
MDMB-5′Cl-BUTINACA	MDMB	Cl	Butyl	110	108–112	0.02	10.6	9.26–12.0	0.03
MDMB-5′Br-BUTINACA	MDMB	Br	Butyl	114	110–117	0.04	42.4	35.3–51.0	0.05
ADB-5′Br-PINACA	ADB	Br	Pentyl	108	106–110	0.04	19.8	17.3–22.6	> 1.00
ADB-4en-5′Br-PINACA	ADB	Br	Pent-4-enyl	111	109–114	0.02	14.2	12.2–16.6	0.20
ADB-5′Br-FUBINACA	ADB	Br	Fluorobenzyl	112	109–116	0.06	42.1	34.6–51.2	0.06
ADB-5′Br-DECINACA	ADB	Br	Decyl	4.89^a^	–	–	ND	ND	–
ADB-5′Br-INACA	ADB	Br	No tail	111	107–115	0.10	2290	1960–2670	< 0.01
MDMB-4en-PINACA	MDMB	–	Pent-4-enyl	109	107–111	0.02	17.9	15.6–20.5	0.90
MDMB-4en-5′Br-PINACA	MDMB	Br	Pent-4-enyl	120	117–123	0.01	44.6	37.8–53.7	< 0.01
MDMB-INACA	MDMB	–	No tail	112	108–115	0.07	3690	3230–4220	< 0.01
MDMB-5′Br-INACA	MDMB	Br	No tail	110	107–112	0.04	2200	1970–2460	< 0.01
MDMB-5′Me-INACA	MDMB	Methyl	No tail	113	107–119	0.28	1370	1050–1790	< 0.01

Abbreviations: ND, not determined (values could not be calculated as saturation was not reached)

^a^Maximal activation observed at a concentration of 60 μM. Accompanying EC_50_ values could not be calculated accurately

**Fig. 2:**
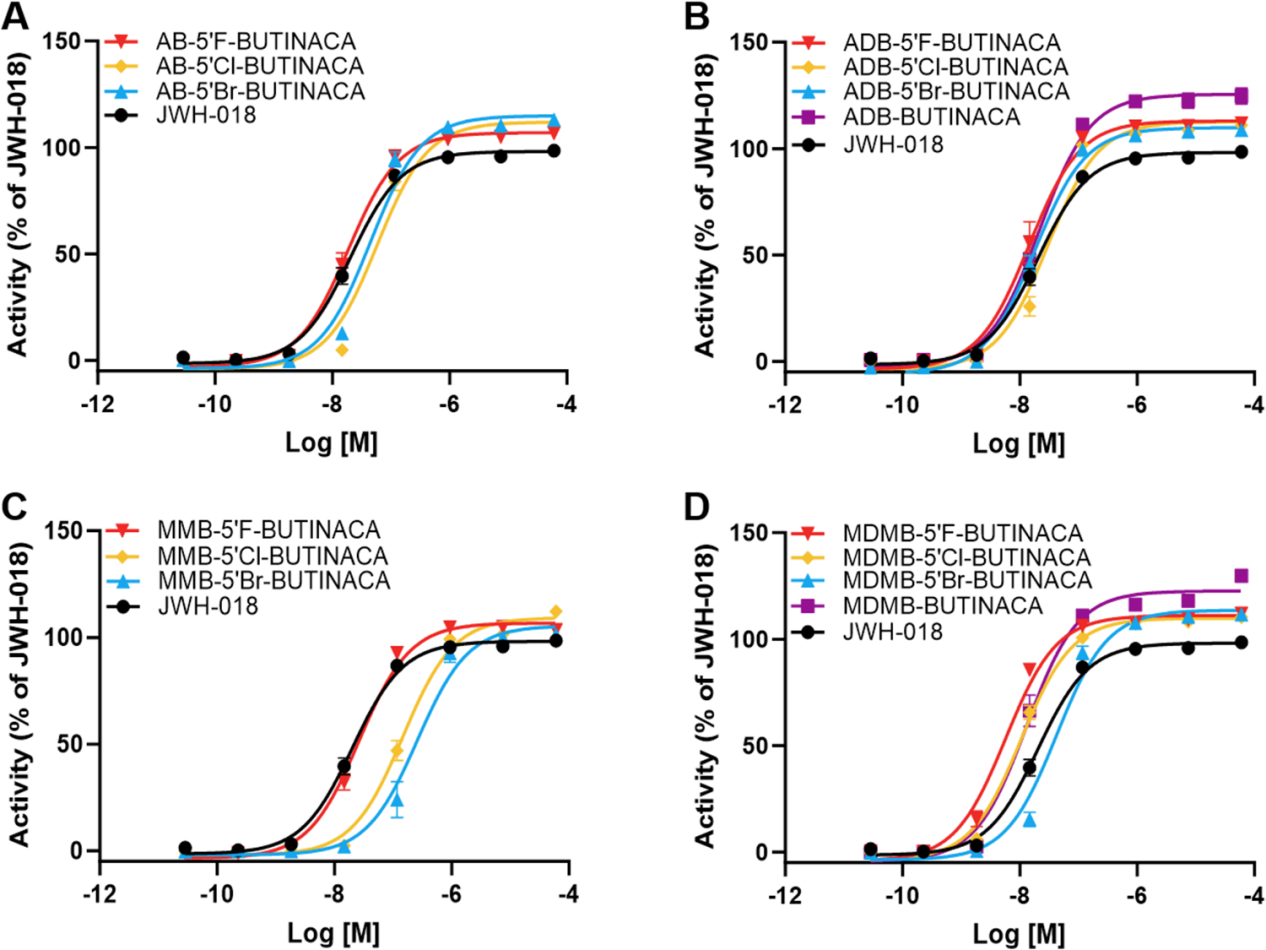
Concentration–response curves for the activation of the CB_1_ receptor by 12 SCRAs with a halogen at the 5 position on the indazole core and two of the non-halogenated analogs: **A** valinamide (AB) SCRAs, **B** *tert*-leucinamide (ADB) SCRAs, **C** valine methyl ester (MMB) SCRAs, and **D** *tert-*leucine methyl ester (MDMB) SCRAs. JWH-018 was used as a reference. Error bars indicate standard error to the mean (SEM)

**Fig. 3:**
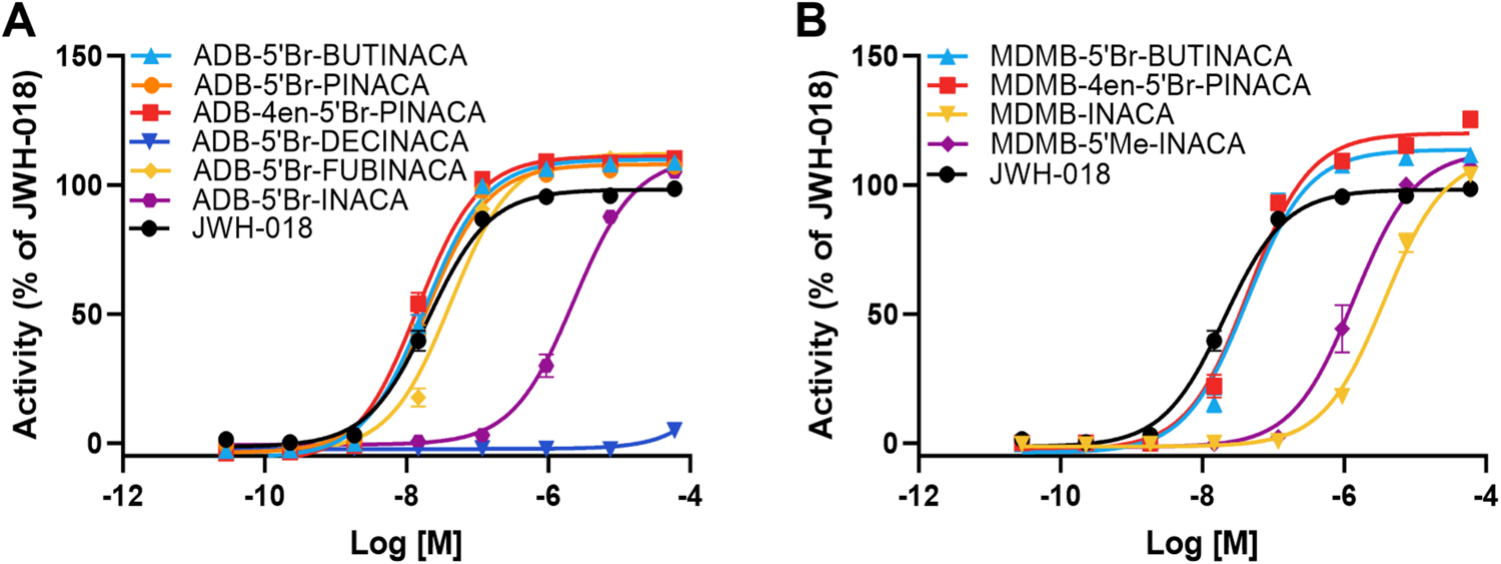
Concentration–response curves for the activation of the CB_1_ receptor by **A** six *tert*-leucinamide SCRAs with a bromine at the 5 position on the indazole core and different tail moieties and **B** four *tert*-leucine methyl ester SCRAs with a bromine or methyl group at the 5 position on the indazole core and different tail moieties. MDMB-INACA is included for comparison of the *tert*-leucine methyl ester SCRAs and JWH-018 is included as a reference for both. Error bars indicate SEM

The dose–response curves of the 12 halogenated indazole SCRAs with a butyl tail are provided alongside the available non-halogenated analogs ADB-BUTINACA and MDMB-BUTINACA in Fig. 2. Unfortunately, reference standards of the non-halogenated analogs AB-BUTINACA and MMB-BUTINACA are not currently available. The dose–response curves for the additional *tert*-leucinamide and *tert*-leucine methyl ester SCRAs with different tail moieties are provided in Fig. 3.

#### Potency

The EC_50_ values of the 24 tested SCRAs ranged from 5.75 to 3690 nM. Ten SCRAs had lower EC_50_ values than JWH-018 (20.6 nM), but only four were significantly lower (*p* < 0.03): MDMB-5′Cl-BUTINACA (10.6 nM), MDMB-BUTINACA (8.90 nM), MDMB-5′F-BUTINACA (5.75 nM), and MDMB-4en-PINACA (17.9 nM). Eight SCRAs had significantly higher EC_50_ values than JWH-018, and MMB-5′F-BUTINACA (26.4 nM) and ADB-5′Cl-BUTINACA (30.1 nM) were found to be not significantly different (*p* = 0.4–0.5).

**Fig. 4:**
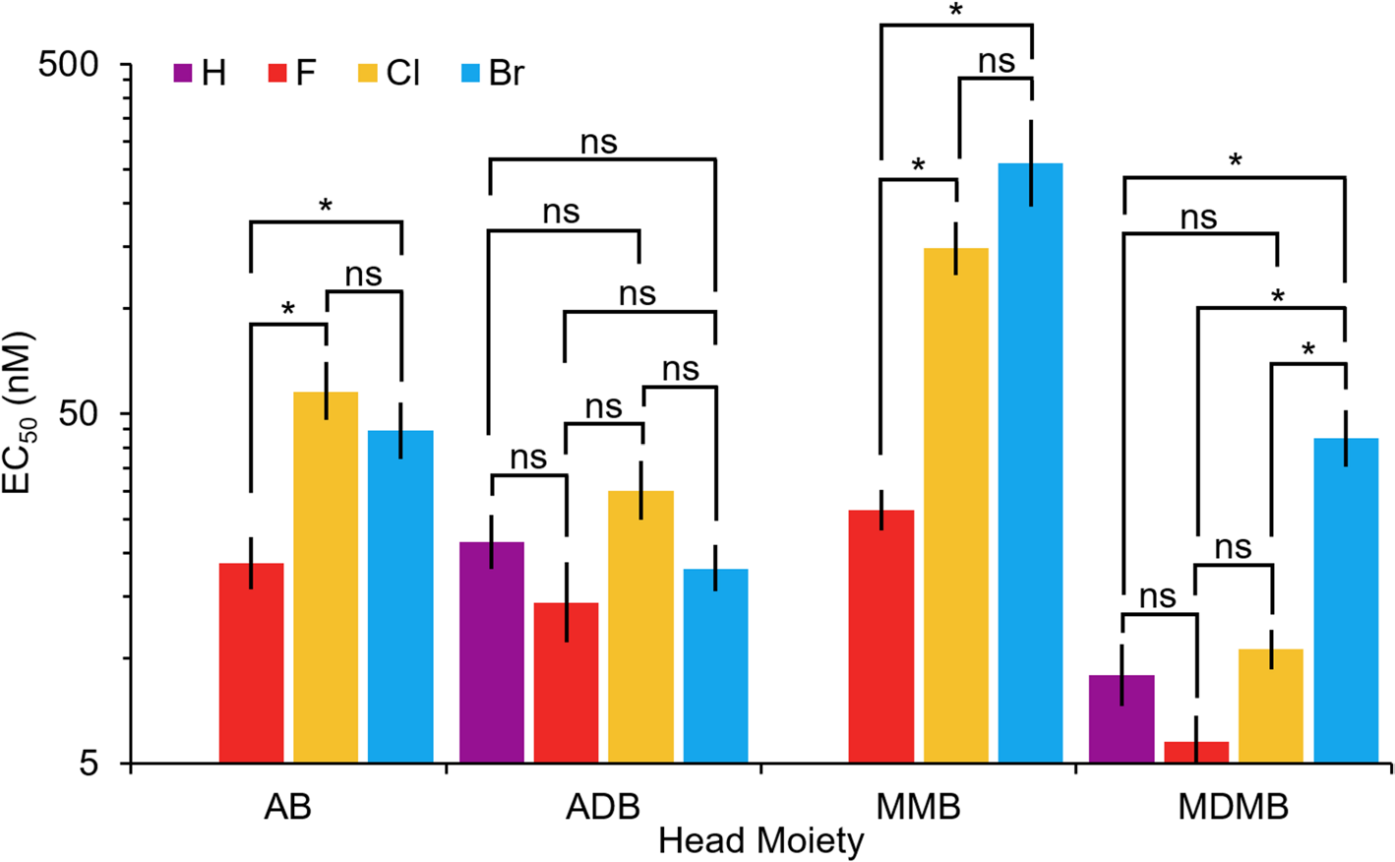
EC_50_ values (nM) on a logarithmic scale of the activation of the CB_1_ receptor by 12 SCRAs with a halogen at the 5 position on the indazole core, including valinamide (AB), *tert-*leucinamide (ADB), valine methyl ester (MMB), and *tert-*leucine methyl ester (MDMB) analogs, and the available non-halogenated analogs. Errors bars represent the 95% confidence interval. The results of statistical comparisons from Brown–Forsythe and Welch ANOVA tests (*α* = 0.05) between the different halogen analogs for each head group are provided where * indicates statistically significant and “ns” indicates not statistically significant

An examination of the SARs of the potencies of different halogen (fluorine, chlorine, or bromine) substitutions revealed that for the valine methyl ester (MMB) and *tert*-leucine methyl ester (MDMB) SCRAs, fluorine was the most potent, being 5.6- and 1.8-fold more potent than chlorine, which was 1.75- and 4.0-fold more potent than the bromine, respectively, as can be seen in Fig. 4 (see the Supplementary Information (Table S1) for complete statistical data). However, two of the relationships were not found to be statistically significant: Br versus Cl for MMB (*p* = 0.1) and F versus Cl for MDMB (*p* = 0.06). This indicates that the potency of methyl ester SCRAs generally increases as the size (van der Waals radius) and mass of the halogen decreases to be more similar to that of hydrogen and electronegativity increases. In comparison to the non-halogenated analog for the *tert*-leucine methyl ester SCRAs, MDMB-5′F-BUTINACA and MDMB-5′Cl-BUTINACA had EC_50_ values similar to MDMB-BUTINACA (F vs. H: *p* = 0.3; Cl vs. H: *p* = 0.9), but MDMB-5′Br-BUTINACA was 4.8-fold less potent (Br vs. H: *p* < 0.01). MDMB-4en-5′Br-BUTINACA was also found to be 2.5-fold less potent (Br vs. H: *p* = 0.01) than its non-halogenated analog MDMB-4en-PINACA. This demonstrates that only bromination at the indazole core significantly reduces the potency for *tert*-leucine methyl ester SCRAs, while the potency of SCRAs with a fluorine and chlorine at the indazole core have comparable potencies to the non-halogenated analog. No previous studies have examined these compounds, so in future, to confirm these SARs, these compounds should be tested on another CB_1_ assay using a different intrinsic signaling pathway, such as a β-arrestin 2 or cyclic AMP (cAMP) assays.

For the valinamide (AB) SCRAs, SARs showed that the fluorinated analog was also the most potent, being 2.4- and 3.1-fold more potent than the brominated and chlorinated, respectively (*p* < 0.01 for F vs. Br and F vs. Cl). However, the brominated was found to be 1.3-fold more potent than the chlorinated, although this relationship was not statistically significant (*p* = 0.4). The *tert-*leucinamide (ADB) SCRAs also showed this relationship, with fluorinated the most potent (1.3- and 2.1-fold more potent than Br Cl, respectively), followed by brominated, which was 1.7-fold more potent than the chlorinated; however, none of the *tert*-leucinamide relationships were significant (F vs. Br: *p* = 0.7; F vs. Cl: *p* = 0.06; Br vs. Cl: *p* = 0.09). These findings are supported by a study using a β-arrestin 2 recruitment assay where ADB-5′F-BUTINACA was also found to be more potent than ADB-5′Br-BUTINACA but with overlapping 95% confidence intervals, indicating the relationship is likely not significant. Unfortunately, the valinamide SCRAs and ADB-5′Cl-BUTINACA were not tested (Deventer et al. 2023b). In comparison to the non-halogenated analog ADB-BUTINACA, ADB-5′F-BUTINACA and ADB-5′Br-BUTINACA were 1.5- and 1.2-fold more potent, respectively (H vs. F: *p* = 0.3; H vs. Br: *p* = 0.7), while ADB-5′Cl-BUTINACA was 1.4-fold less potent (H vs. Cl: p = 0.3). Although ADB-BUTINACA was found to be more potent than ADB-5′F-BUTINACA and ADB-5′Br-BUTINACA on the β-arrestin 2 recruitment assay (Deventer et al. 2023b; Sparkes et al. 2022), no statistical analysis was performed in the studies and the 95% confidence interval for ADB-BUTINACA was not available for comparison; therefore, it is not possible to evaluate the statistical significance of this relationship. Overall, this demonstrates that halogenation at the 5 position on the indazole core does not significantly affect the potency for *tert*-leucinamide SCRAs and *tert*-leucinamide SCRAs are the least affected by halogenation at the indazole core in comparison to other head moieties.

When the SCRAs are tail-less, such as with MDMB-INACA, a bromine on the indazole core (MDMB-5′Br-INACA) was 1.7-fold more potent, although the difference was not statistically significant (H vs. Br: *p* = 0.06). The newly emerged SCRA MDMB-5′Me-INACA with a methyl group at the 5 position on the indazole core was found to be the most potent of the three tail-less *tert*-leucine methyl ester SCRAs, being 2.7- and 1.6-fold more potent than MDMB-INACA (H vs. Me: *p* = 0.07) and MDMB-5′Br-INACA (Me vs. Br: *p* = 0.4). This indicates that similar to the SCRAs with a butyl tail, bromination and methylation does not significantly change the potency. SCRAs with a butyl tail and methyl group at the 5 position on the indazole core are not currently available, but should be examined in future to determine if the addition of a methyl group at the 5 position on the indazole core also demonstrates no significant change in potency as seen with the tail-less SCRAs.

Of the different head moieties, valine methyl ester (MMB) SCRAs were the least potent, followed by valinamide (AB) (1.4- to 5.8-fold more potent than MMB), *tert*-leucinamide (ADB) (1.3- to 2.5-fold more potent than AB), and *tert*-leucine methyl ester (MDMB) (> 1.0- to 2.8-fold more potent than ADB); however, when the indazole core was brominated, the *tert*-leucine methyl ester was 2.4- to 3.1-fold less potent than the *tert*-leucinamide. It should be noted that with regards to the statistical tests, of the 19 comparisons of SCRA analogs with different head groups, all but three (AB-5′F-BUTINACA vs. MMB-5′F-BUTINACA: *p* = 0.2; AB-5′F-BUTINACA vs. ADB-5′F-BUTINACA: *p* = 0.6; AB-5′Br-BUTINACA vs. MDMB-5′Br-BUTINACA: *p* = 1) were found to be significant (see Supplementary Information (Table S2) for more details). From this data, it is clear the *tert*-butyl group on the head moiety causes increased potency compared to the isopropyl group; however, due to the inconsistency in the SAR between the *tert*-leucinamide and *tert*-leucine methyl ester SCRAs, the relationship between the formamide (ADB) and methyl formate (MDMB) groups on the head moiety is less certain. These results are consistent with findings from the examination of the binding of MDMB-FUBINACA using the crystal structure of the CB_1_ receptor that the *tert-*butyl moiety is important for potency and the methyl formate is important for binding in the receptor (Kumar et al. 2019). The SARs of the head moieties identified in this study are also similar to findings from other studies that systematically evaluated the in vitro activity of indazole SCRAs using a fluorescence-based imaging plate reader (FLIPR) membrane potential assay and a live cell-based β-arrestin 2 recruitment assay (Banister and Connor 2018; Grafinger et al. 2021; Noble et al. 2019), which demonstrates consistent results across three different in vitro CB_1_ assays.

When considering the core halogenated *tert*-leucinamide and *tert*-leucine methyl ester (ADB and MDMB) SCRAs included in this study, it is possible to compare the effect of the different tail moieties on potency. Among the brominated SCRAs, pent-4-enyl (ADB-4en-5′Br-PINACA; MDMB-4en-5′Br-PINACA), butyl (ADB-5′Br-BUTINACA; MDMB-5′Br-BUTINACA), and pentyl (ADB-5′Br-PINACA) SCRAs were all within a 1.4-fold difference of each other, so not significantly different (ADB pent-4-enyl vs. butyl: *p* = 0.6; ADB pent-4-enyl vs. pentyl: *p* = 0.2; ADB butyl vs. pentyl: *p* = 1.0; MDMB pent-4-enyl vs. butyl: *p* = 1.0). Of the remaining examined tails, fluorobenzyl (ADB-5′Br-FUBINACA) was the next most potent, being 2.3-fold less potent than the butyl (*p* = 0.03), followed by the tail-less (ADB-5′Br-INACA), which was 127-fold less potent than the butyl (*p* < 0.01), and the decyl alkyl chain (ADB-5′Br-DECINACA). The decyl chain was by far the least potent of these compounds with ADB-5′Br-DECINACA not even reaching saturation to allow an EC_50_ value to be calculated, as can clearly be seen in Fig. 3A. Due to its lipophilicity, it is possible the solubility of ADB-5′Br-DECINACA in the aqueous assay medium may have contributed to the lack of activity; however, similar to all the other SCRAs tested, no precipitation of the compound was observed in the dilution series, indicating its dissolution. The tail-less analogs studied, ADB-5′Br-INACA and MDMB-5′Br-INACA, were 100-fold weaker in potency than JWH-018. This has been observed for other tail-less SCRAs, including ADB-INACA, which did not reach saturation in either a β-arrestin 2 recruitment or Ca^2+^ release assay, so EC_50_ values were unable to be calculated (Deventer et al. 2023b). These results are in agreement with previous studies using FLIPR and β-arrestin 2 recruitment assays that have found a four to six carbon alkyl chain is the best for potency and efficacy with reduced potency found for shorter and longer alkyl chains, as well as ring structures like the fluorobenzyl (Banister and Connor 2018; Grafinger et al. 2021; Krotulski et al. 2021).

#### Efficacy

The maximal receptor activity (*E*_max_) of the 24 tested SCRAs ranged from 105 to 121%. Only one SCRA (MMB-5′Br-BUTINACA) had a 95% confidence interval (CI) that overlapped with JWH-018, but only 12 of the tested SCRAs were found to have a significantly greater *E*_max_ than JWH-018 (99.7%) as can be seen in Table 1.

When examining possible SARs, no significant differences in efficacy were observed between the different halogen (fluorine, chlorine, or bromine) substitutions on the indazole core. Halogenated *tert*-leucinamide and *tert*-leucine methyl ester SCRAs had 1.1-fold lower efficacy than their non-halogenated analog ADB-BUTINACA (H vs. F: *p* = 0.04; H vs. Cl: *p* = 0.02; H vs. Br; *p* < 0.01) and MDMB-BUTINACA (H vs. F: *p* = 0.07; H vs. Cl: *p* = 0.07; H vs. Br; *p* = 0.3), respectively. There were no significant differences in efficacy between the different head moieties (AB, ADB, MMB, or MDMB) (*p* = 0.09 to > 1). Finally, there were no significant differences in efficacy between the different tail moieties (*p* = 0.3 to > 1); however, the efficacy of ADB-5′Br-DECINACA was unable to be determined due to a lack of saturation as clearly shown in Fig. 3A. The lack of significant differences for the efficacy between SCRAs is likely due to the clustering of the efficacies, which has been previously identified as a limitation of AequoScreen^®^ assays (Charlton and Vauquelin 2010; Deventer et al. 2023b).

## Conclusion

This study is the first to systematically evaluate the effect on the in vitro CB_1_ receptor activity of the addition of a halogen (fluorine, bromine, or chlorine) at the 5 position on the indazole core of SCRAs using 19 different halogenated SCRAs. Overall, of SCRAs with a substitution at the 5 position on the indazole core, analogs with a fluorine were found to result in the lowest EC_50_ values. For SCRAs with a methyl ester head moiety (MMB or MDMB), analogs with a chlorine had the next lowest EC_50_ values followed by brominated analogs; however, this relationship was the opposite for SCRAs with an amide (AB or ADB) head moiety. *Tert*-leucinamide (ADB) SCRAs were the least affected by halogenation of the indazole core with all compounds having similar potency to each other and the non-halogenated analog ADB-BUTINACA. The halogenated *tert-*leucine methyl ester (MDMB) SCRAs also had similar potency to their non-halogenated analogs (MDMB-BUTINACA and MDMB-4en-PINACA), apart for the brominated analogs (MDMB-5′Br-BUTINACA and MDMB-4en-5′Br-PINACA, respectively), which had significantly reduced potency. In future, in order to confirm the SARs identified in this study, the compounds should be tested on another CB_1_ assay using a different intrinsic signaling pathway, such as a β-arrestin 2 or cyclic AMP (cAMP). They should also be examined on a CB_2_ assay to better understand their overall cannabimimetic activity.

In addition, the newly emerged SCRA with a methyl group at the 5 position on the indazole core (MDMB-5′Me-INACA) was not found to be significantly different from its non-halogenated analog, MDMB-INACA, or brominated analog, MDMB-5′Br-INACA. This indicates methylation also does not lead to significant changes in potency or efficacy; however, this relationship should be examined further with more methylated SCRAs, such as those with an alkyl chain tail.

## Supplementary Information

Below is the link to the electronic supplementary material.

Supplementary file1 (DOCX 1237 KB)

## Data Availability

All data supporting the findings of this study are available within the paper and its Supplementary Information.
