# Supplementary material for: In vitro CB1 receptor activity of halogenated indazole synthetic cannabinoid receptor agonists
Source: Arch Toxicol. 2025 May 18;99(8):3343–53. doi: 10.1007/s00204-025-04082-4 (PMC12367851; doi:10.1007/s00204-025-04082-4)
Supplement: Supplementary file 1 — Supplementary file1 (DOCX 1237 KB) [file 204_2025_4082_MOESM1_ESM.docx]

***In vitro* CB_1_ receptor activity of halogenated indazole synthetic cannabinoid receptor agonists**

Henrik Green^1,2^, Craig McKenzie^3^, Elias Hamra^1^, Tobias Rautio^4^, Xiongyu Wu^4^, Emma Juneskog^1^, Rebecka Sandblom^1^, Manuela Carla Monti^1^, Mattias Persson^1,2^, Caitlyn Norman^1,*^

^1^Department of Biomedical and Clinical Science, Division of Clinical Chemistry and Pharmacology, Linköping University, Linköping, Sweden

^2^Department of Forensic Genetics and Forensic Toxicology, National Board of Forensic Medicine, Linköping, Sweden

^3^Chiron AS, Trondheim, Norway

^4^Department of Physics, Chemistry and Biology, Linköping University, Linköping, Sweden

^*^Corresponding author: caitlyn.norman@liu.se

**Supplementary Information**

**Section 1: Synthesis schemes of SCRA reference standards**

**Section 2: Analytical characterization of synthesized SCRA reference standards**

**Section 3: Structure-activity relationship statistical analysis results**

**Section 1: Synthesis schemes of SCRA reference standards**


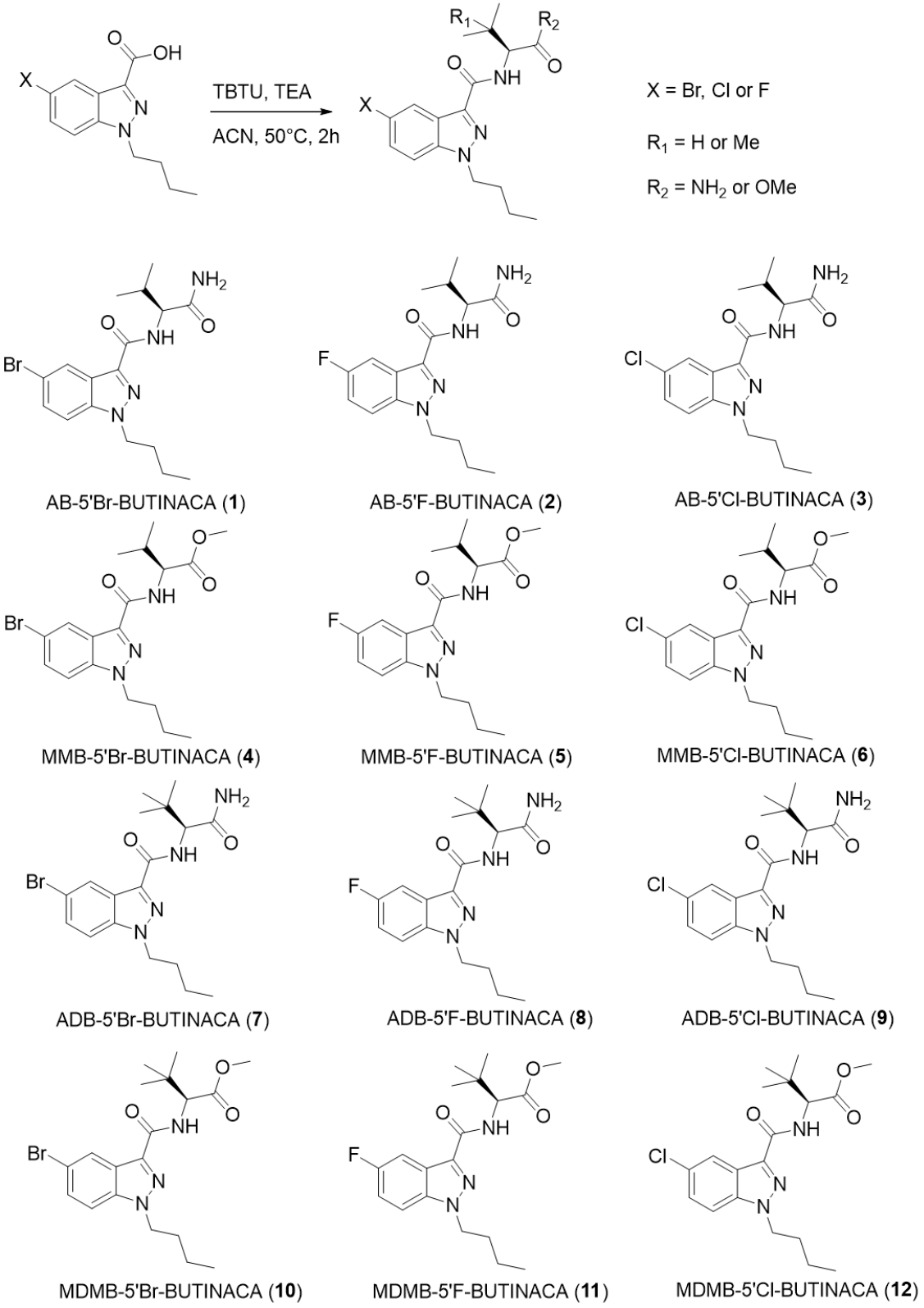


**Figure S1.1.** Synthesis of ADB-5’Br-BUTINACA, MDMB-5’Br-BUTINACA, AB-5’Br-BUTINACA, MMB-5’Br-BUTINACA, ADB-5’Cl-BUTINACA, MDMB-5’Cl-BUTINACA, AB-5’Cl-BUTINACA, MMB-5’Cl-BUTINACA, ADB-5’F-BUTINACA, MDMB-5’F-BUTINACA, AB-5’F-BUTINACA, and MMB-5’F-BUTINACA.


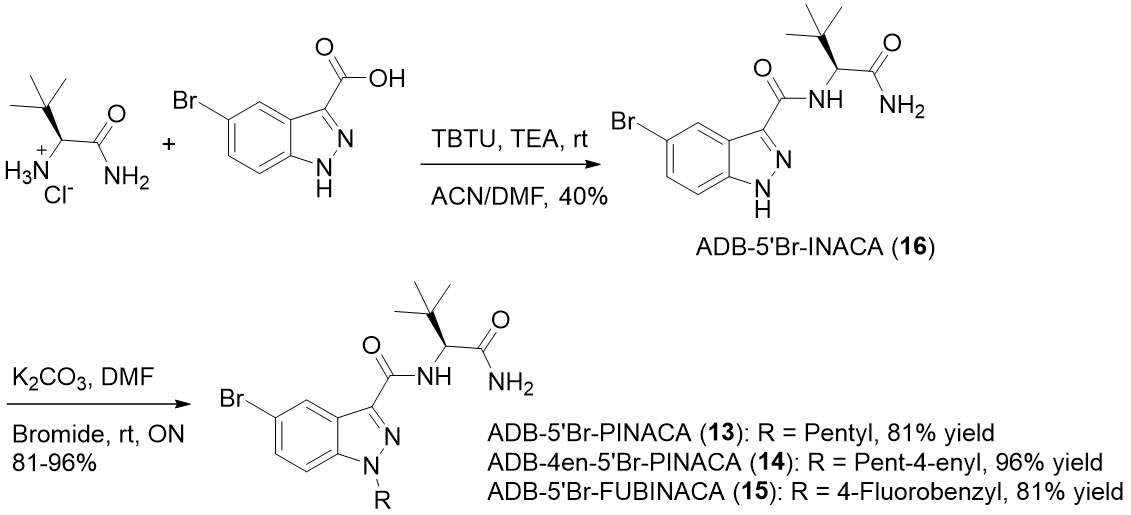


**Figure S1.2.** Synthesis of ADB-5’Br-PINACA, ADB-4en-5’Br-PINACA, and ADB-5’Br-FUBINACA.

**Section 2: Analytical characterization of synthesized SCRA reference standards**

**Table S2.1.** NMR data of the sixteen synthesized SCRAs.

| **SCRA** | **^1^H-NMR (500 MHz), δ in ppm** | **^13^C-NMR (126 MHz), δ in ppm** |
| --- | --- | --- |
| AB-5’Br-BUTINACA | (chloroform-*d*) 8.44 (d, *J* = 1.0 Hz, 1H), 7.51 (br d, *J* = 9.0 Hz, 1H, NH), 7.45 (dd, *J* = 9.0, 1.0 Hz, 1H), 7.28 (d, *J* = 9.0 Hz, 1H), 6.69 (br s, 1H, NH_2_), 6.02 (br s, 1H, NH_2_), 4.59 (dd, *J* = 8.5, 7.0 Hz, 1H), 4.34 (t, *J* = 7.0 Hz, 2H), 2.31 (octet, *J* = 6.5 Hz, 1H), 1.89 (quintet, *J* = 6.5 Hz, 2H), 1.32 (sextet, *J* = 7.5 Hz, 2H), 1.08 (d, *J* = 7.5 Hz, 3H), 1.06 (d, *J* = 7.0 Hz, 3H), 0.93 (t, *J* = 7.5 Hz, 3H). | (chloroform-*d*) 173.9, 162.5, 139.6, 135.9, 130.0, 125.1, 124.3, 116.2, 110.9, 57.9, 49.6, 31.8, 31.0, 20.1, 19.6, 18.4, 13.7. |
| AB-5’F-BUTINACA | (chloroform-*d*) 7.90 (dd, *J* *=* 8.5, 2.0 Hz, 1H), 7.51 (br d, *J* = 9.0 Hz, 1H, NH), 7.35 (dd, *J* = 9.0, 4.0 Hz, 1H), 7.16 (dt, *J =* 9.0, 2.0 Hz, 1H), 6.68 (br s, 1H, NH_2_), 5.99 (br s, 1H, NH_2_), 4.59 (dd, *J* = 8.5, 7.5 Hz, 1H), 4.36 (t, *J* = 7.0 Hz, 2H), 2.32 (octet, *J* = 7.0 Hz, 1H), 1.90 (quintet, *J* = 7.5 Hz, 2H), 1.34 (sextet, *J* = 7.5 Hz, 2H), 1.08 (d, *J* = 6.5 Hz, 3H), 1.06 (d, *J* = 6.5 Hz, 3H), 0.94 (t, *J* = 7.0 Hz, 3H). | (chloroform-*d*) 173.9, 162.8, 159.1 (d, *J_CF_ =* 241.1 Hz), 137.9, 136.4 (d, *J_CF_ =* 5.8 Hz), 123.2 (d, *J_CF_ =* 8.3 Hz), 116.6 (d, *J_CF_ =* 28.0 Hz), 110.7 (d, *J_CF_ =* 9.7 Hz), 106.7 (d, *J_CF_ =* 24.7 Hz), 57.9, 49.7, 31.9, 30.9, 20.1, 19.6, 18.3, 13.7. |
| AB-5’Cl-BUTINACA | (chloroform-*d*) 8.27 (d, *J* = 1.0 Hz, 1H), 7.51 (br d, *J* = 9.0 Hz, 1H, NH), 7.33 (s, 2H), 7.28 (d, *J* = 9.0 Hz, 1H), 6.66 (br s, 1H, NH_2_), 6.00 (br s, 1H, NH_2_), 4.63-4.56 (m, 1H), 4.35 (t, *J* = 7.0 Hz, 2H), 2.32 (octet, *J* = 7.5 Hz, 1H), 1.90 (quintet, *J* = 7.5 Hz, 2H), 1.33 (sextet, *J* = 7.5 Hz, 2H), 1.08 (d, *J* = 7.0 Hz, 3H), 1.07 (d, *J* = 7.0 Hz, 3H), 0.94 (t, *J* = 7.5 Hz, 3H). | (chloroform-*d*) 173.8, 162.6, 139.4, 136.0, 128.6, 127.6, 123.7, 121.9, 110.6, 57.9, 49.6, 31.8, 31.0, 20.1, 19.6, 18.4, 13.7. |
| MMB-5’Br-BUTINACA | (chloroform-*d*) 8.52 (s, 1H), 7.45 (dd, *J* = 8.5, 1.0 Hz, 1H), 7.42 (br d, *J* = 9.0 Hz, 1H, NH), 7.28 (d, *J* = 9.0 Hz, 1H), 4.77 (dd, *J* = 9.0, 5.0 Hz, 1H), 4.36 (t, *J* = 7.0 Hz, 2H), 3.78 (s, 3H), 2.30 (octet, *J* = 6.5 Hz, 1H), 1.91 (quintet, *J* = 7.5 Hz, 2H), 1.34 (sextet, *J* = 7.5 Hz, 2H), 1.04 (d, *J* = 7.5 Hz, 3H), 1.02 (d, *J* = 8.0 Hz, 3H), 0.95 (t, *J* = 7.5 Hz, 3H). | (chloroform-*d*) 172.6, 162.2, 139.6, 136.1, 130.0, 125.4, 124.4, 116.2, 110.8, 56.9, 52.3, 49.6, 31.8, 31.7, 20.1, 19.3, 18.1, 13.7. |
| MMB-5’F-BUTINACA | (chloroform-*d*) 7.96 (d, *J* *=* 9.0 Hz, 1H), 7.42 (br d, *J* = 9.0 Hz, 1H, NH), 7.35 (dd, *J* = 9.0, 3.5 Hz, 1H), 7.15 (dt, *J =* 9.0, 1.5 Hz, 1H), 4.77 (dd, *J* = 9.0, 5.0 Hz, 1H), 4.37 (t, *J* = 7.0 Hz, 2H), 3.77 (s, 3H), 2.30 (octet, *J* = 6.5 Hz, 1H), 1.91 (quintet, *J* = 7.5 Hz, 2H), 1.35 (sextet, *J* = 7.5 Hz, 2H), 1.04 (d, *J* = 7.0 Hz, 3H), 1.02 (d, *J* = 7.5 Hz, 3H), 0.95 (t, *J* = 7.5 Hz, 3H). | (chloroform-*d*) 172.7, 162.4, 159.1 (d, *J_CF_ =* 241.2 Hz), 137.9, 136.6 (d, *J_CF_ =* 5.8 Hz), 123.3 (d, *J_CF_ =* 11.0 Hz), 116.6 (d, *J_CF_ =* 28.0 Hz), 110.6 (d, *J_CF_ =* 9.8 Hz), 107.0 (d, *J_CF_ =* 24.7 Hz), 56.8, 52.3, 49.6, 31.9, 31.7, 20.1, 19.2, 18.1, 13.7. |
| MMB-5’Cl-BUTINACA | (chloroform-*d*) 8.34 (t, *J* *=* 1.5 Hz, 1H), 7.42 (br d, *J* = 9.0 Hz, 1H, NH), 7.33 (d, *J* = 1.5 Hz, 2H), 4.78 (dd, *J* = 9.0, 5.0 Hz, 1H), 4.37 (t, *J* = 7.0 Hz, 2H), 3.78 (s, 3H), 2.35-2.25 (m, 1H), 1.91 (quintet, *J* = 7.5 Hz, 2H), 1.35 (sextet, *J* = 7.5 Hz, 2H), 1.04 (d, *J* = 7.0 Hz, 3H), 1.02 (d, *J* = 7.0 Hz, 3H), 0.95 (t, *J* = 7.5 Hz, 3H). | (chloroform-*d*) 172.7, 162.2, 139.4, 136.2, 128.6, 127.6, 123.8, 122.1, 110.5, 56.9, 52.3, 49.6, 31.9, 31.7, 20.1, 19.3, 18.1, 13.7. |
| ADB-5’Br-BUTINACA* | (chloroform-*d*) 8.43 (s, 1H), 7.67 (d, *J* = 9.5 Hz, 1H), 7.45 (d, *J* = 8.9 Hz, 1H), 7.29 (d, *J* = 8.9 Hz, 1H) 6.71 (bs, 1H), 5.93 (bs, 1H) 4.68 (d, *J* = 9.5 Hz, 1H), 4.36 (t, *J* = 7.2 Hz, 2H), 1.90 (q, *J* = 7.4 Hz, 2H), 1.33 (h, *J* = 7.5 Hz, 2H), 1.15 (s, 9H), 0.94 (t, *J* = 7.4 Hz, 3H). | (chloroform-*d*) 173.0, 162.3, 139.6, 136.0, 129.9, 125.1, 124.3, 116.2, 110.9, 59.7, 49.6, 34.8, 31.9, 26.8, 20.1, 13.7. |
| ADB-5’F-BUTINACA* | (chloroform-*d*) 7.86 (dd, *J* = 8.8, 2.0 Hz, 1H), 7.68 (d, *J* = 9.5 Hz, 1H), 7.35 (dd, *J* = 9.1, 3.9 Hz, 1H) 7.15 (td, *J* = 8.9, 2.3 Hz, 1H), 6.84 (bs, 1H), 6.03 (bs, 1H), 4.70 (d, *J* = 9.6 Hz, 1H), 4.36 (t, *J* = 7.2 Hz, 2H), 1.90 (q, *J* = 7.4 Hz, 2H), 1.34 (h, *J* = 7.5 Hz, 2H), 1.14 (s, 9H), 0.94 (t, *J* = 7.4 Hz, 3H). | (chloroform-*d*) 173.2, 162.5, 159.5 (*J_CF_* = 240.6 Hz CF-aromatic), 137.9, 136.4 (*J_CF_* = 5.7 Hz C-aromatic), 123.1 (*J_CF_* = 11.2 Hz C-aromatic), 116.5 (*J_CF_* = 28.0 Hz CH-aromatic), 110.6 (*J_CF_* = 9.7 Hz C-aromatic), 106.6 (*J_CF_* = 24.5 Hz CH-aromatic), 59.7, 49.7, 34.8, 31.9, 26.8, 20.1, 13.7 |
| ADB-5’Cl-BUTINACA* | (chloroform-*d*) 8.25 (s, 1H), 7,67 (d, *J* = 9.5 Hz, 1H), 7.33 (s, 2H), 6.71 (bs, 1H), 5.93 (bs, 1H) 4.69 (d, *J* = 9.6 Hz, 1H), 4.36 (t, *J* = 7.2 Hz, 2H), 1.90 (q, *J* = 7.4 Hz, 2H), 1.33 (h, *J* = 7.5 Hz, 2H), 1.15 (s, 9H), 0.95 (t, *J* = 7.4 Hz, 3H). | (chloroform-*d*) 173.0, 162.3., 139.4, 136.1, 128.6, 127.5, 123.7.1, 121.8, 59.7, 49.7, 34.8, 31.9, 26.9, 20.1, 13.7 |
| MDMB-5’Br-BUTINACA* | (chloroform-*d*) 8.52 (s, 1H), 7,50 (d, J = 9.6 Hz, 1H), 7.46 (d, J = 8.9 Hz, 1H) 7.28 (d, J = 8.9 Hz, 1H), 4.70 (d, J = 9.6 Hz, 1H), 4.36 (t, J = 7.2 Hz, 2H), 3.76 (s, 3H), 1.91 (q, J = 7.4 Hz, 2H), 1.35 (h, J = 7.6 Hz, 2H), 1.08 (s, 9H), 0.96 (t, J = 7.3 Hz, 3H). | (chloroform-*d*) 172.2, 162.0, 139.6, 136.1, 130.0, 125.4, 124.4, 116.2, 110.8, 59.6, 51.9, 49.6, 35.2, 31.9, 26.8, 20.1, 13.7. |
| MDMB-5’F-BUTINACA* | (chloroform-*d*) 7.95 (dd, *J* = 8.7, 1.3 Hz, 1H), 7,50 (d, *J* = 9.6 Hz, 1H), 7.34 (dd, *J* = 9.1, 3.9 Hz, 1H) 7.15 (td, *J* = 8.9, 1.6 Hz, 1H), 4.70 (d, *J* = 9.7 Hz, 1H), 4.37 (t, *J* = 7.2 Hz, 2H), 3.75 (s, 3H), 1.91 (q, *J* = 7.4 Hz, 2H), 1.35 (h, *J* = 7.5 Hz, 2H), 1.08 (s, 9H), 0.96 (t, *J* = 7.4 Hz, 3H).  ^19^FNMR (282 MHz): -120.4 | (chloroform-*d*) 172.2, 162.2, 159.1 (*J_C,F_* = 240.6 Hz, CF-aromatic), 137.9, 136.5 (*J_C,F_* = 5.8 Hz, C-aromatic), 123.3 (*J_C,F_* = 11.5 Hz, C-aromatic), 116.6 (*J_C,F_* = 28.1 Hz, CH-aromatic), 110.5 (*J_C,F_* = 9.7 Hz C-aromatic), 107.0 (*J_C,F_* = 24.7 Hz, CH-aromatic), 59.6, 51.9, 49.6, 35.1, 31.9, 26.8, 20.1, 13.7 |
| MDMB-5’Cl-BUTINACA* | (chloroform-*d*) 8.34 (s, 1H), 7,50 (d, *J* = 9.6 Hz, 1H), 7.33 (d, *J* = 1.3 Hz, 2H), 4.70 (d, *J* = 9.7 Hz, 1H), 4.37 (t, *J* = 7.2 Hz, 2H), 3.76 (s, 3H), 1.91 (q, *J* = 7.4 Hz, 2H), 1.35 (h, *J* = 7.5 Hz, 2H), 1.08 (s, 9H), 0.96 (t, *J* = 7.4 Hz, 3H). | (chloroform-*d*) 172.1, 161.9., 139.3, 136.1, 128.5, 127.5, 123.7, 122.0, 110.4, 59.5, 51.8, 49.5, 35.0, 31.8, 26.7, 20.0, 13.6. |
| ADB-5’Br-PINACA | (chloroform-*d*) 8.45 (d, *J* = 1.0 Hz, 1H), 7.67 (br d, *J* = 9.5 Hz, 1H), 7.47 (dd, *J* = 9.0, 1.5 Hz, 1H), 7.29 (d, *J* = 9.0 Hz, 1H), 6.55 (br s, 1H), 5.82 (br s, 1H), 4.66 (d, *J* = 9.0 Hz, 1H), 4.35 (t, *J* = 7.5 Hz, 2H), 1.93 (quin, *J* = 7.5 Hz, 2H), 1.40-1.25 (m, 4H), 1.15 (s, 9H), 0.88 (t, *J* = 7.0 Hz, 3H). | (chloroform-*d*) 173.0, 162.3, 139.7, 136.0, 130.0, 125.2, 124.4, 116.2, 110.9, 59.8, 49.9, 34.8, 29.5, 29.0, 26.9, 22.3, 14.0. |
| ADB-4en-5’Br-PINACA | (chloroform-*d*) 8.44 (s, 1H), 7.68 (br d, *J* = 9.5 Hz, 1H, NH), 7.46 (dd, *J* = 9.0, 1.0 Hz, 1H), 7.28 (d, *J* = 9.0 Hz, 1H), 6.66 (br s, 1H, NH_2_), 5.92 (br s, 1H, NH_2_), 5.84-5.74 (m, 1H, 4en), 5.05 (br d, *J* = 3.5 Hz, 1H, 4en), 5.02 (br s, 1H, 4en), 4.69 (d, *J* = 9.5 Hz, 1H), 4.36 (t, *J* = 6.5 Hz, 2H), 2.12-2.00 (m, 4H), 1.15 (s, 9H). | (chloroform-*d*) 173.0, 162.3, 139.7, 136.9, 136.2, 130.0, 125.1, 124.4, 116.2, 110.9, 59.8, 49.0, 34.8, 30.7, 28.8, 26.9. |
| ADB-5’Br-FUBINACA | (chloroform-*d*) 8.46 (s, 1H), 7.69 (br d, *J* = 9.5 Hz, 1H, NH), 7.41 (dd, *J* = 9.0, 1.0 Hz, 1H), 7.20-7.14 (m, 3H), 6.99 (t, *J =* 8.5 Hz, 2H), 6.53 (br s, 1H), 5.86 (br s, 1H), 5.55 (s, 2H), 4.67 (d, *J* = 9.5 Hz, 1H), 1.15 (s, 9H).  ^19^FNMR (282 MHz): -113.5 | (chloroform-*d*) 172.9, 162.7 (d, *J_CF_ =* 247.7 Hz) 162.1, 139.5, 136.7, 131.3 (d, *J_CF_ =* 3.3 Hz), 130.4, 129.1 (d, *J_CF_ =* 5.8 Hz), 125.3, 124.8, 116.6, 116.0 (d, *J_CF_ =* 21.8 Hz), 111.2, 59.8, 53.4, 34.9, 26.9. |
| ADB-5’Br-INACA | (Acetone-d6) 8.45 (s, 1H), 7.86 (br d, *J* = 9.5 Hz, 1H), 7.64 (d, *J* = 9.0 Hz, 1H), 7.53 (d, *J* = 9.0 Hz, 1H), 7.36 (br s, 1H), 6.81 (br s, 1H), 4.63 (d, *J* = 9.0 Hz, 1H), 1.10 (s, 9H). | (Acetone-d6) 173.4, 162.3, 141.2, 138.9, 130.6, 125.1, 124.3, 116.0, 113.5, 60.2, 35.3, 27.2. |

*indicates data previously reported in Rautio et al, 2024 in Forensic Chemistry.


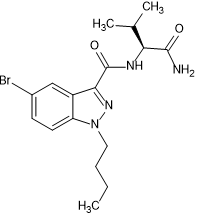

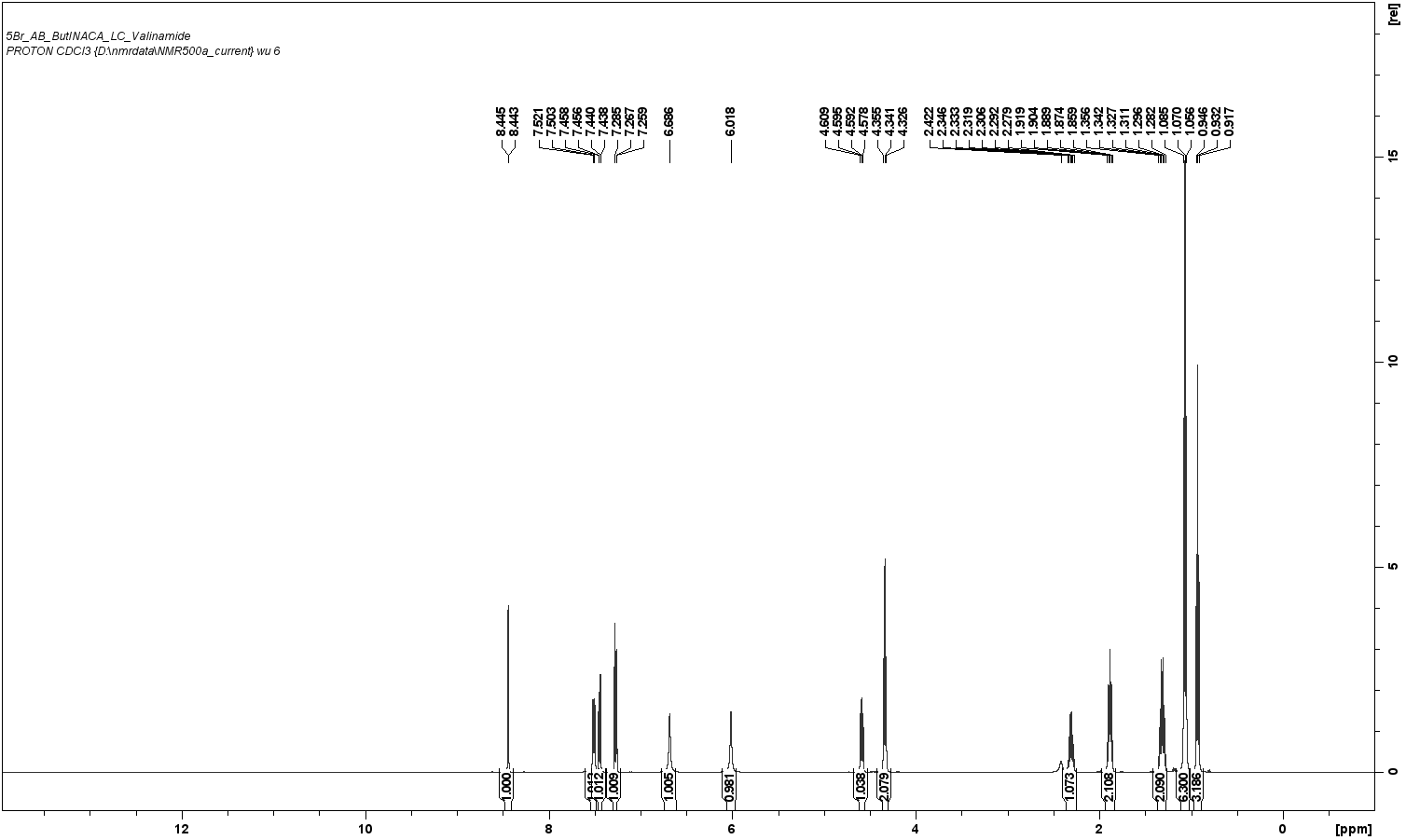


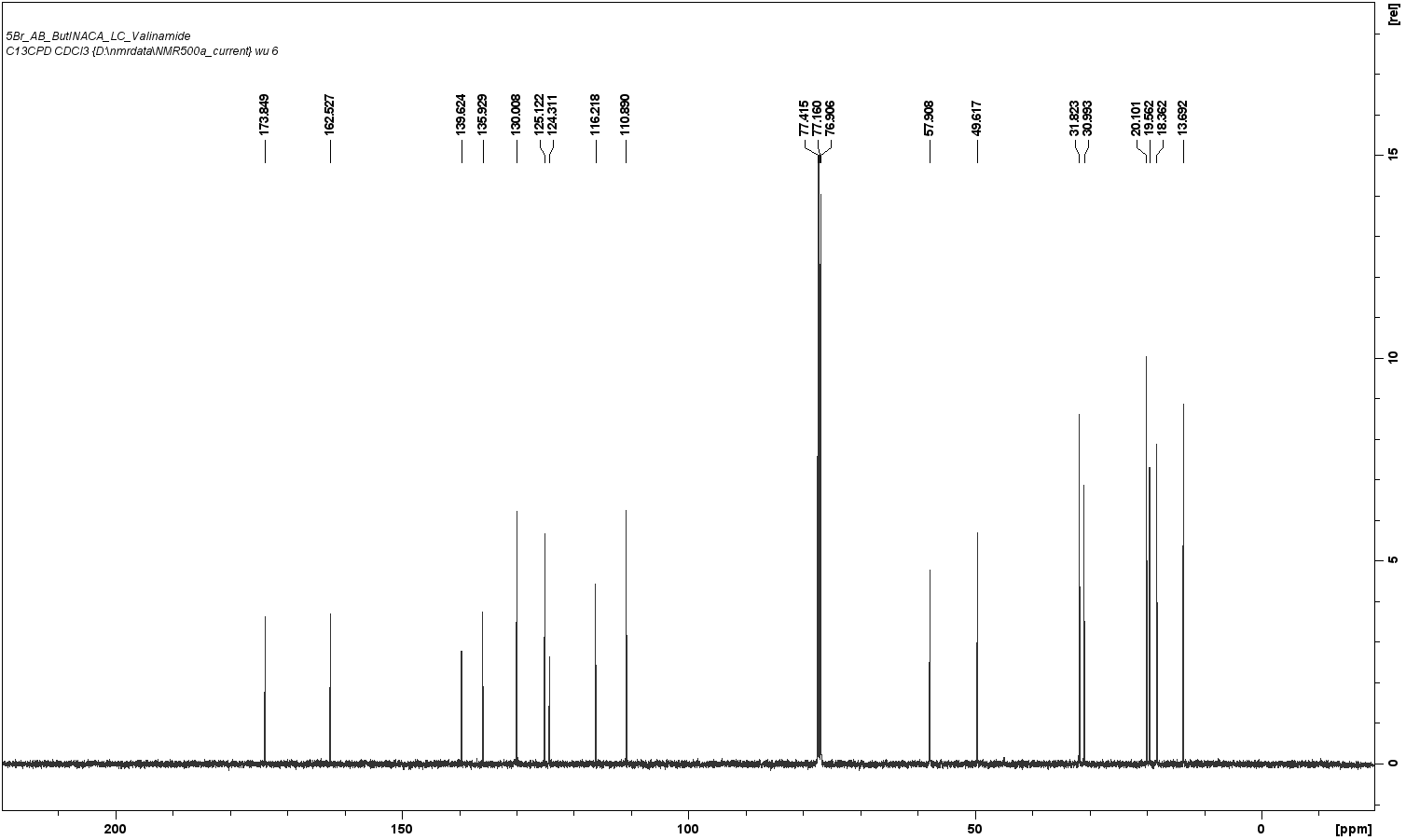
**Figure S2.1.** Chemical structure, ^1^H-NMR (500 MHz) spectrum, and ^13^C-NMR (126 MHz) spectrum for AB-5’Br-BUTINACA.


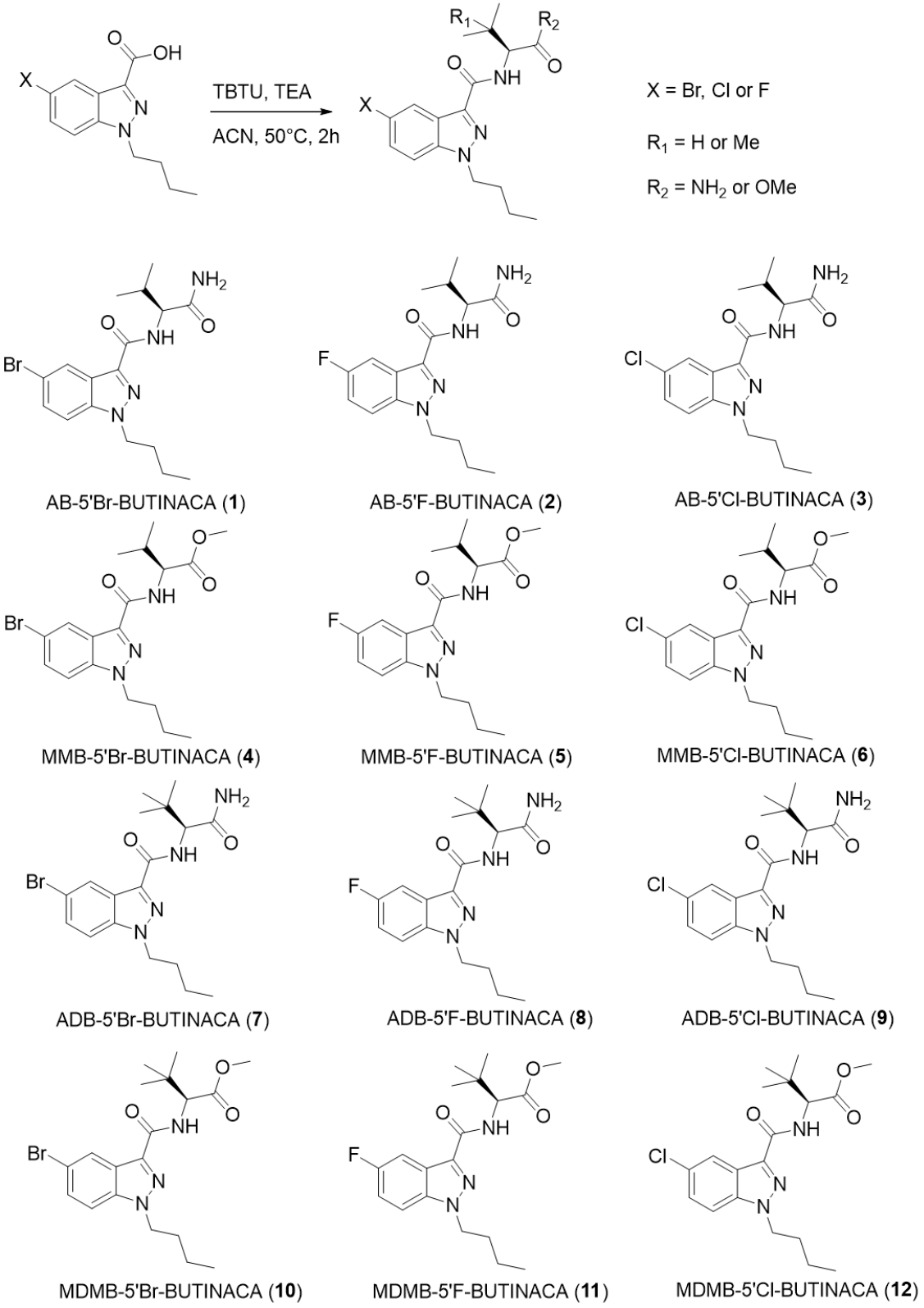

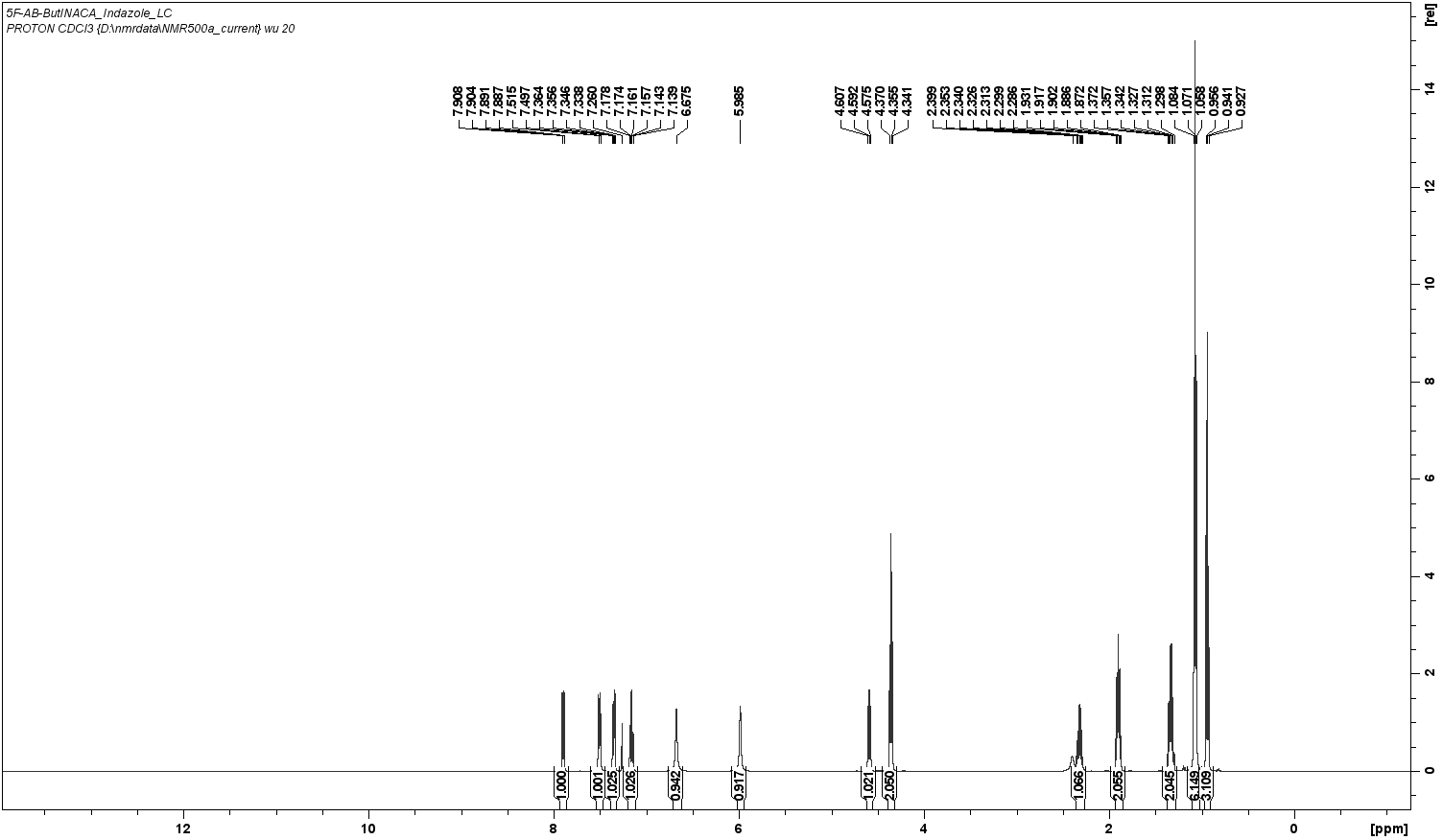


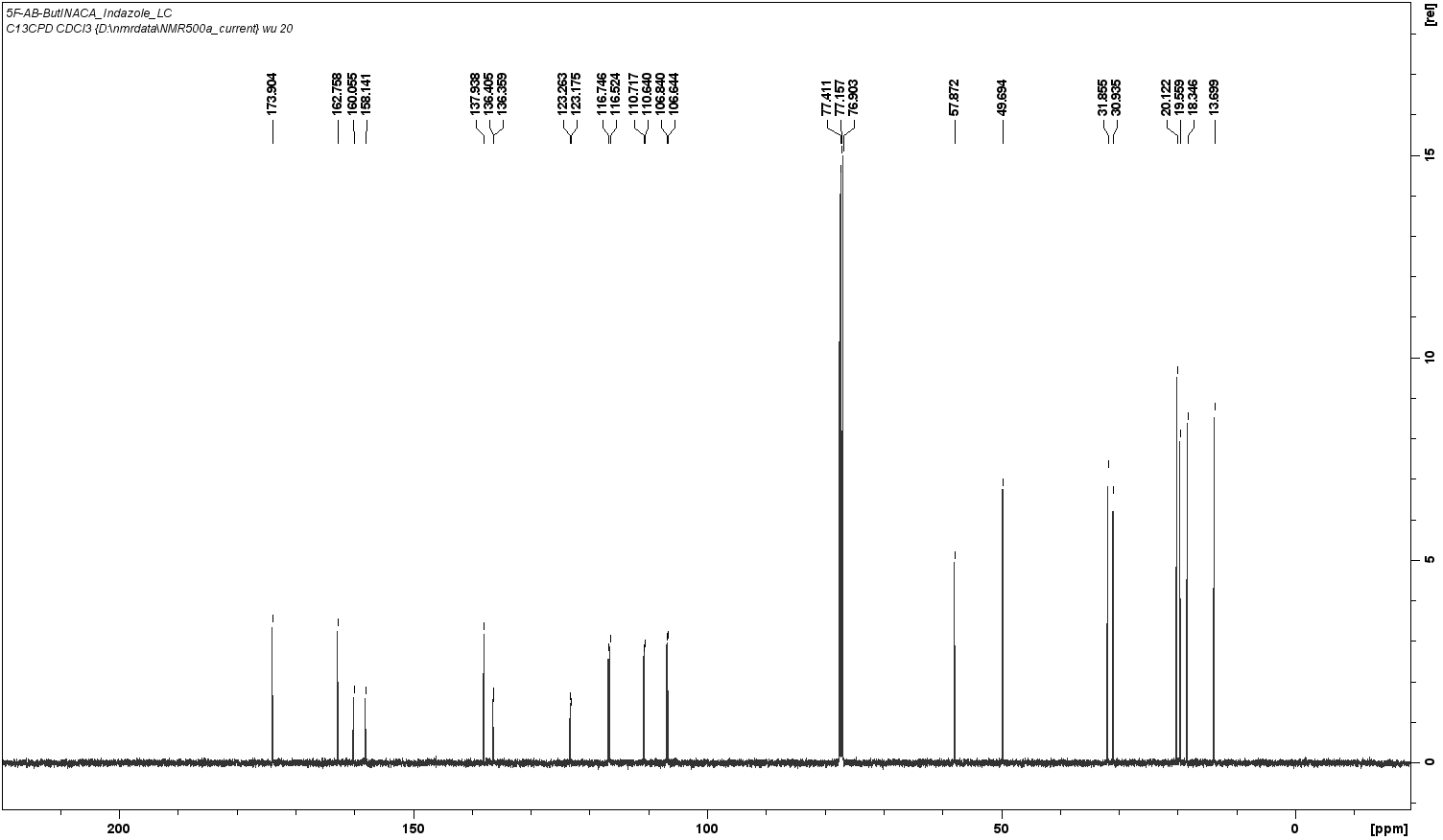
**Figure S2.2.** Chemical structure, ^1^H-NMR (500 MHz) spectrum, and ^13^C-NMR (126 MHz) spectrum for AB-5’F-BUTINACA.


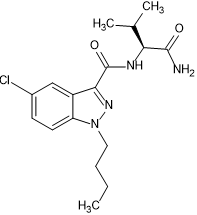

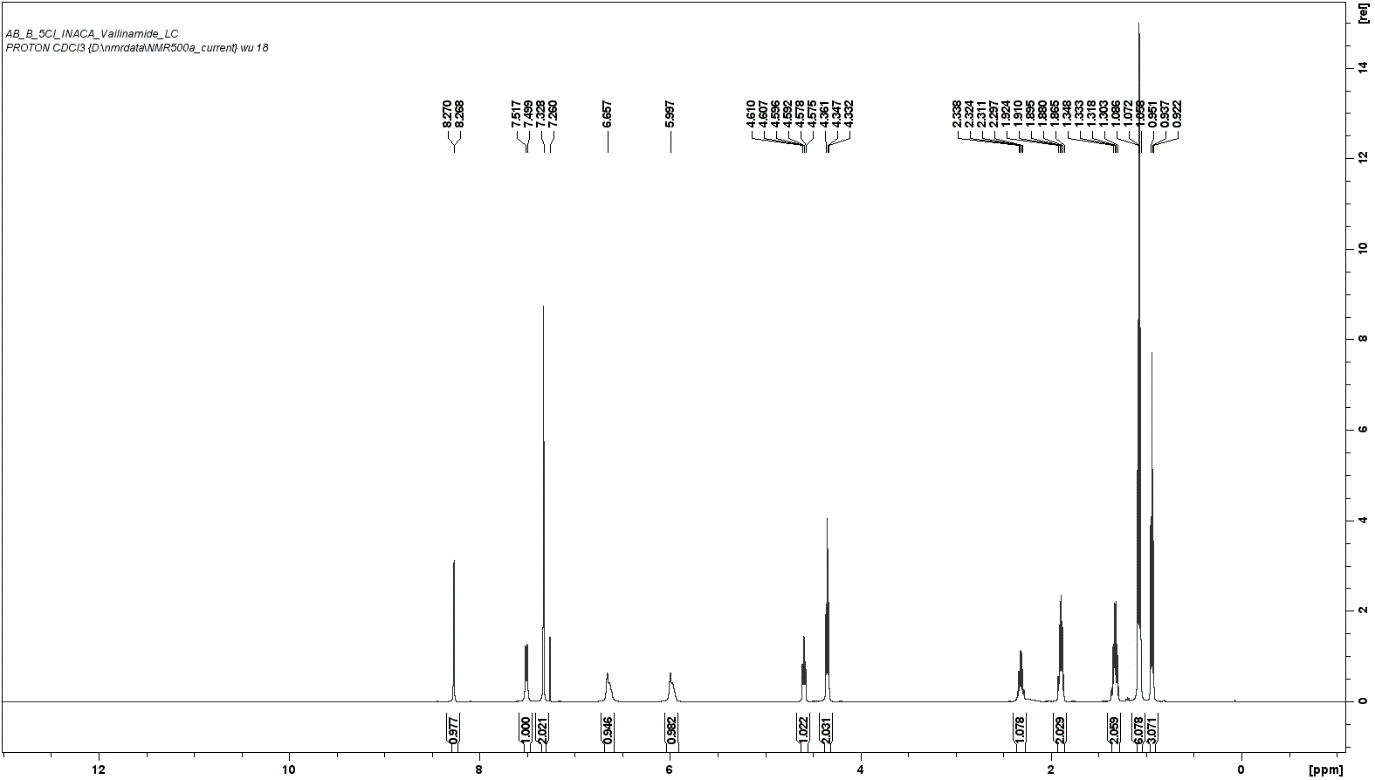


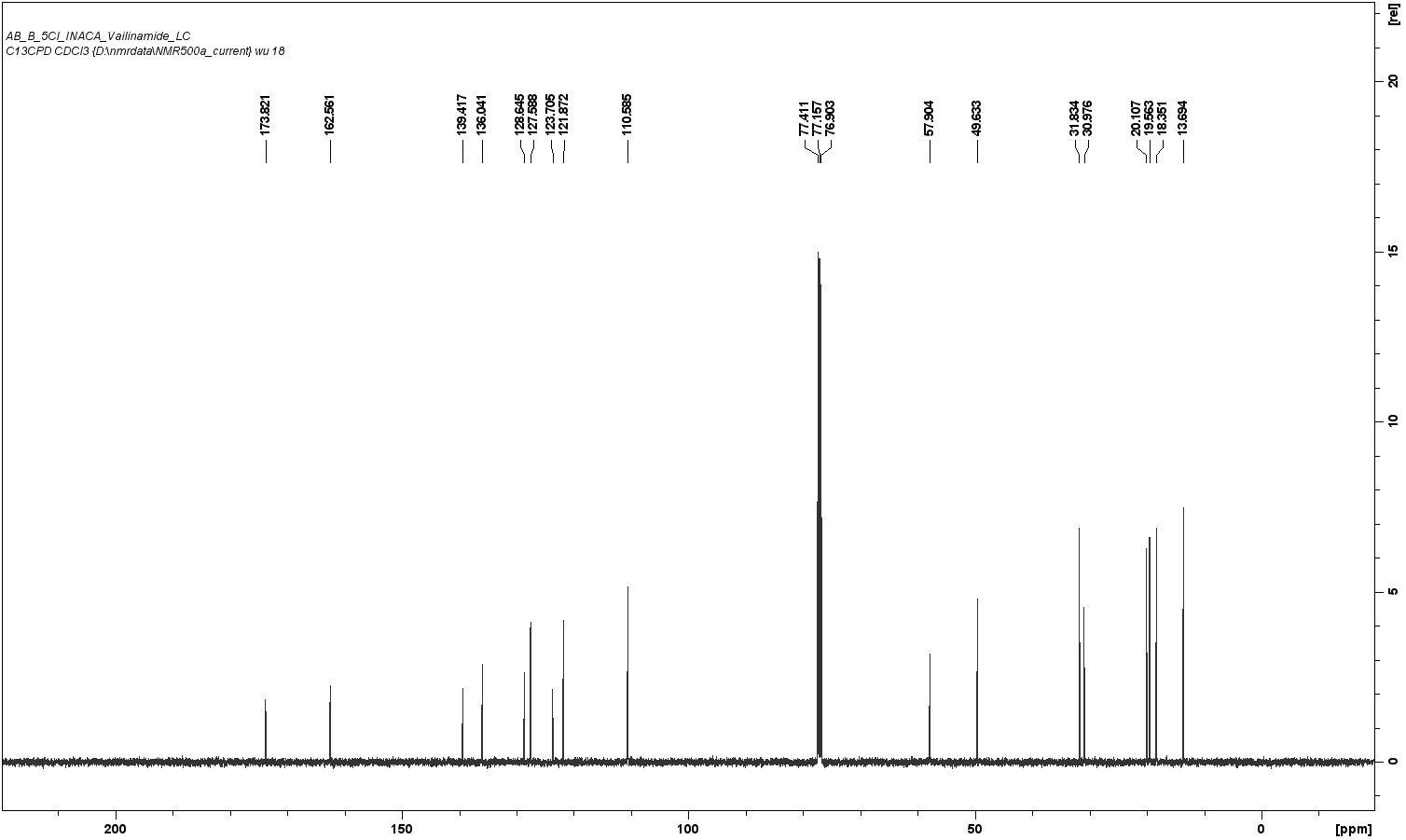
**Figure S2.3.** Chemical structure, ^1^H-NMR (500 MHz) spectrum, and ^13^C-NMR (126 MHz) spectrum for AB-5’Cl-BUTINACA.


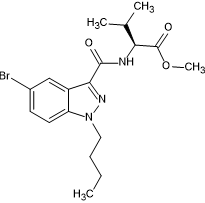

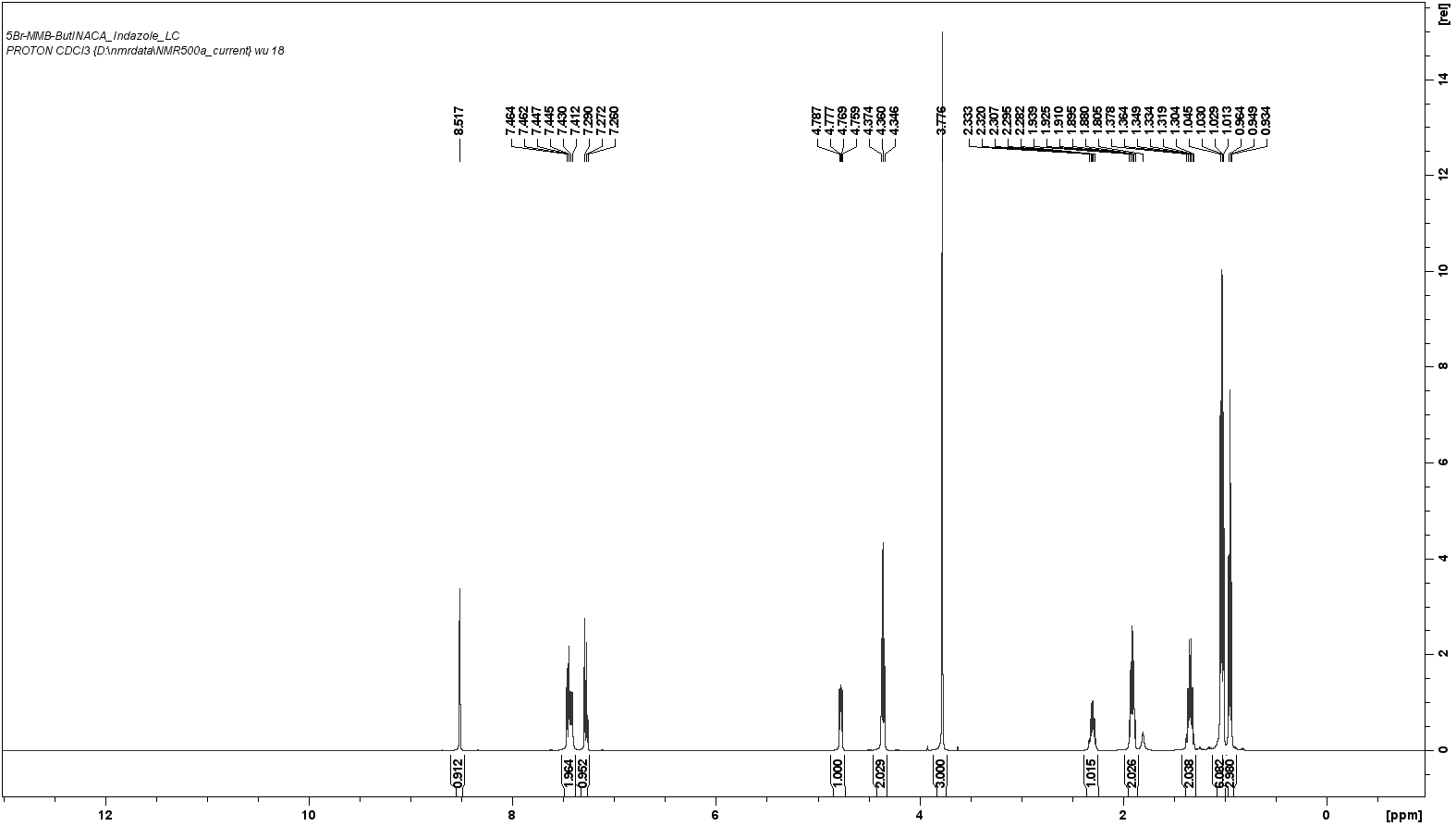


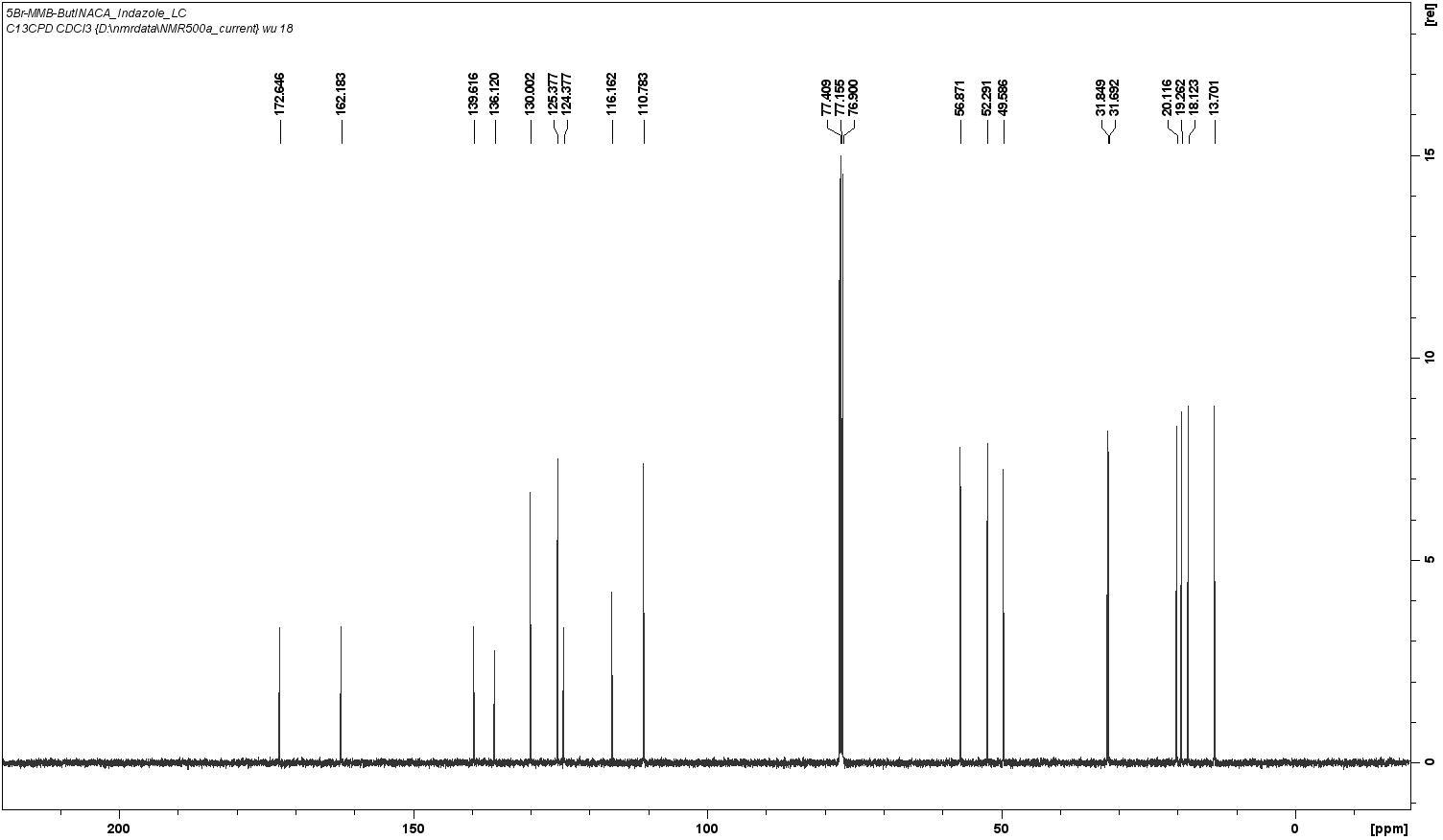
**Figure S2.4.** Chemical structure, ^1^H-NMR (500 MHz) spectrum, and ^13^C-NMR (126 MHz) spectrum for MMB-5’Br-BUTINACA.


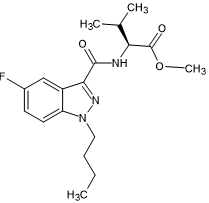

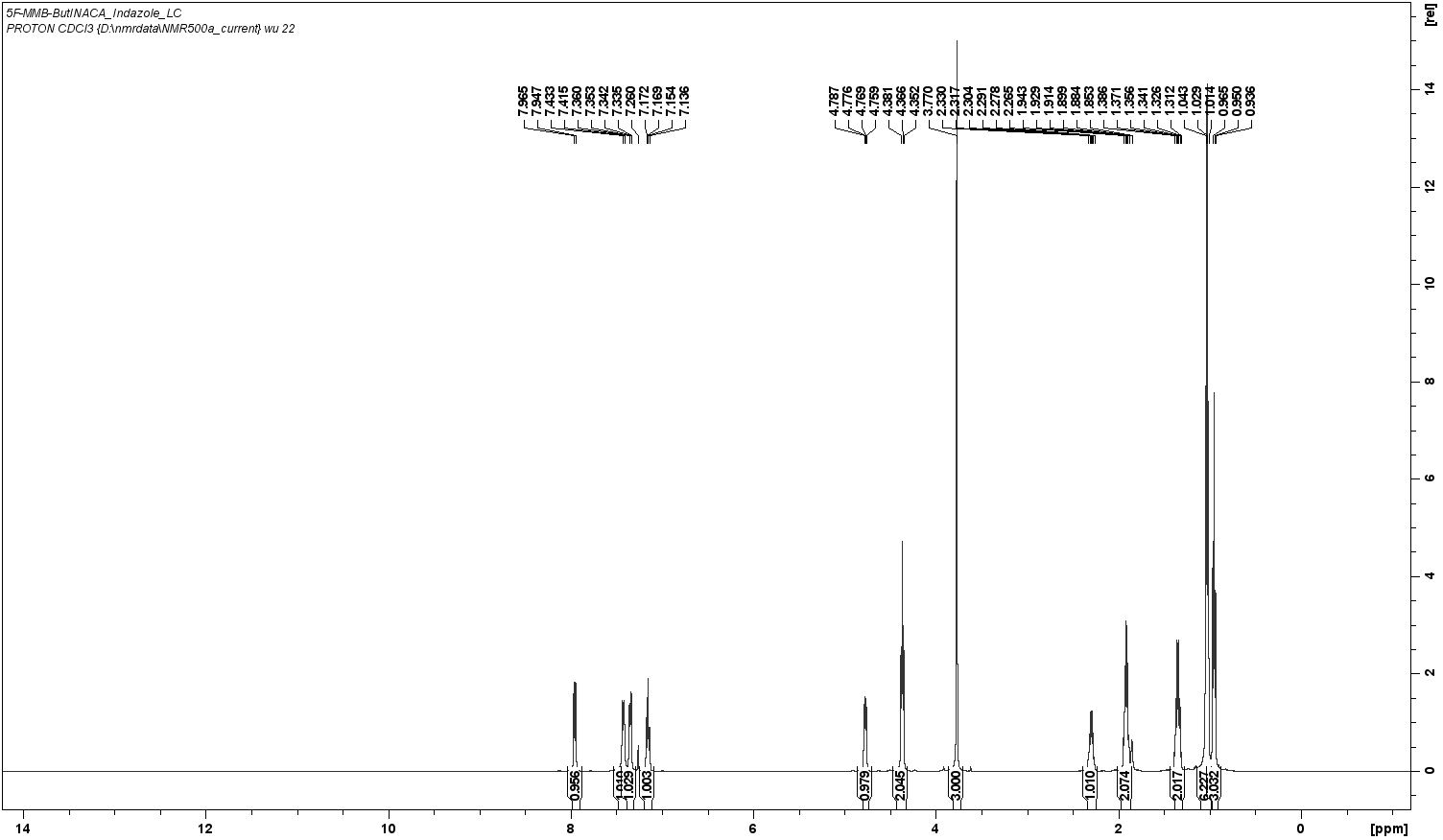


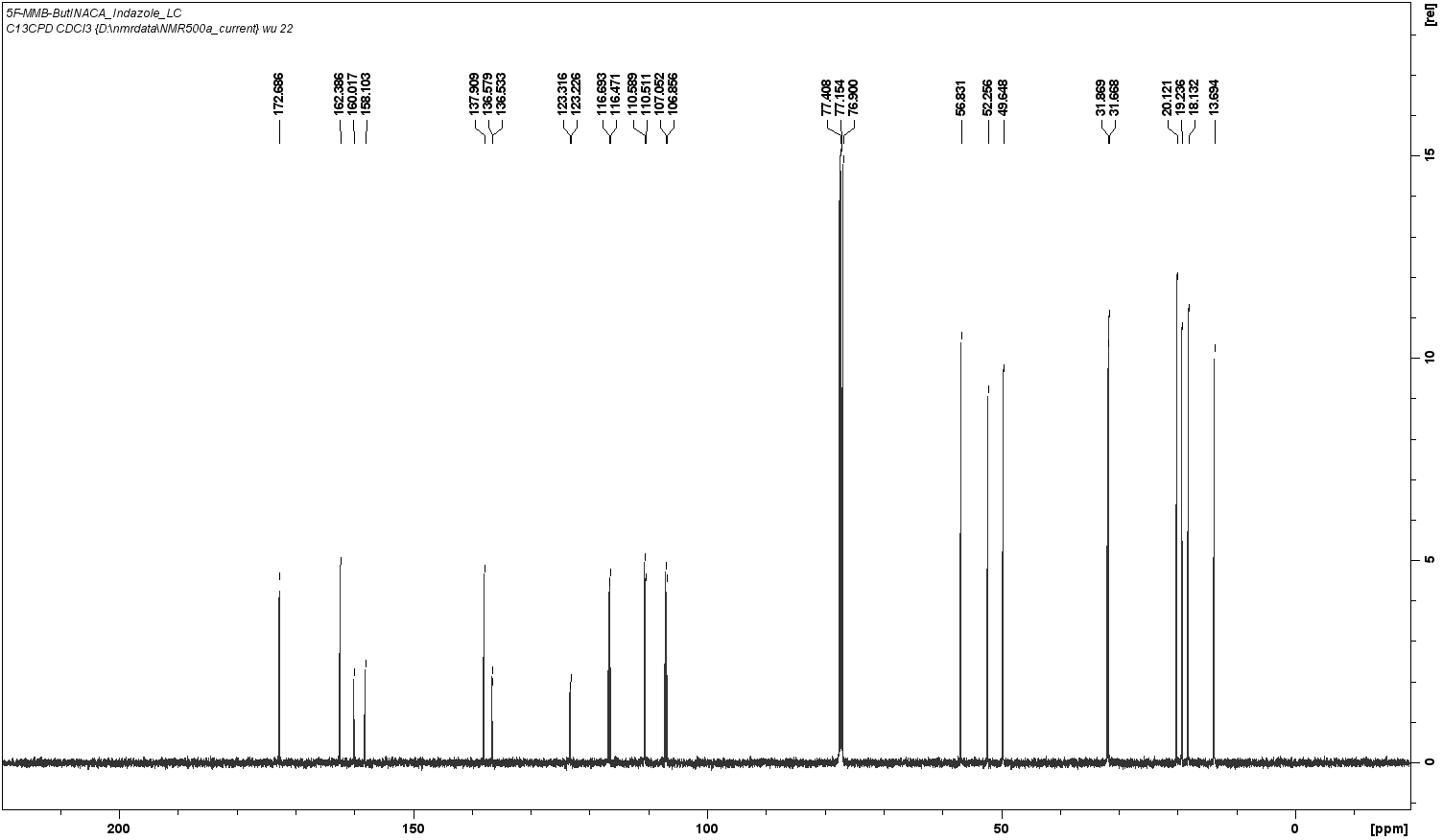
**Figure S2.5.** Chemical structure, ^1^H-NMR (500 MHz) spectrum, and ^13^C-NMR (126 MHz) spectrum for MMB-5’F-BUTINACA.


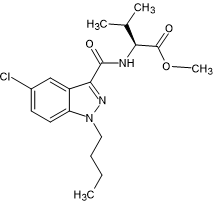

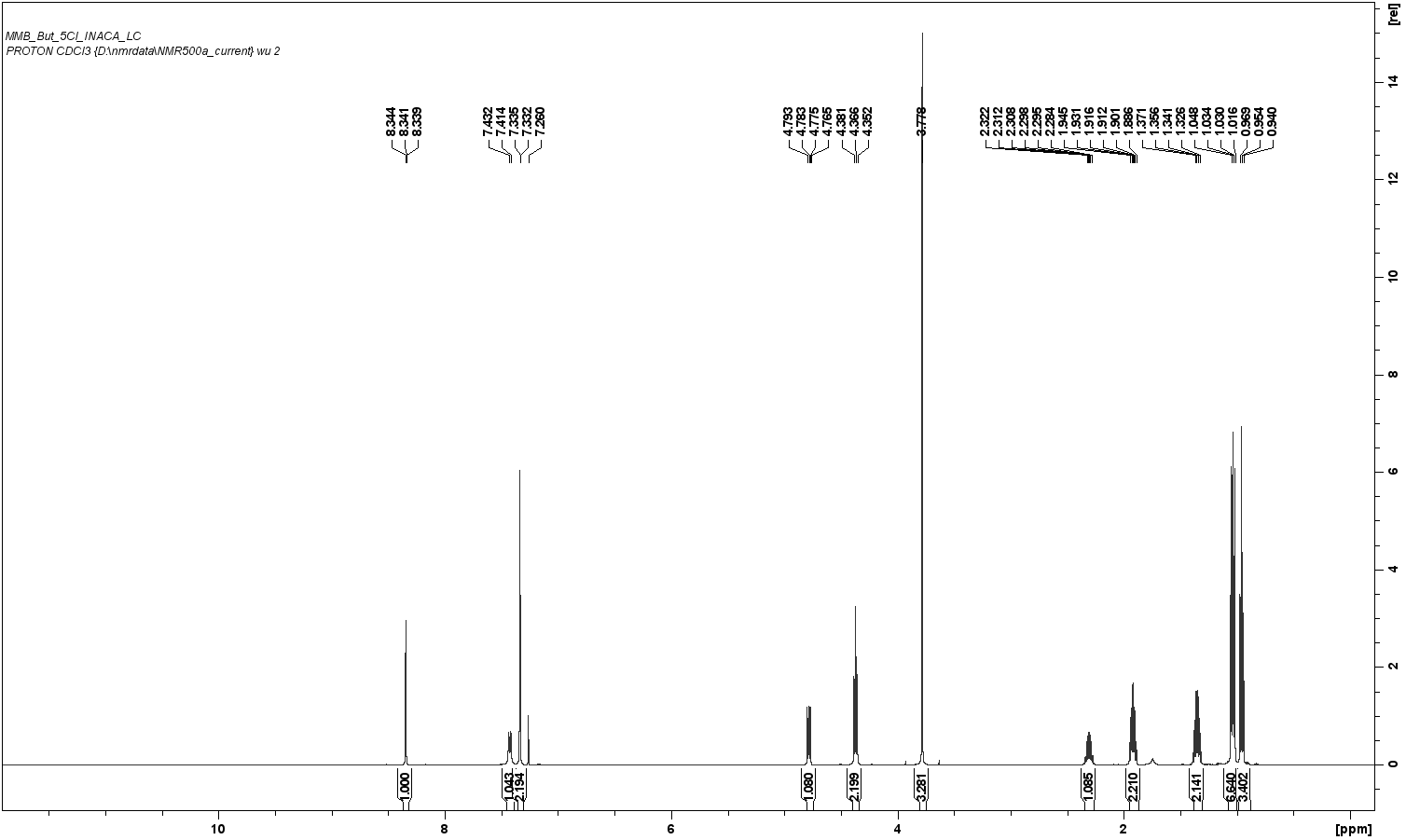


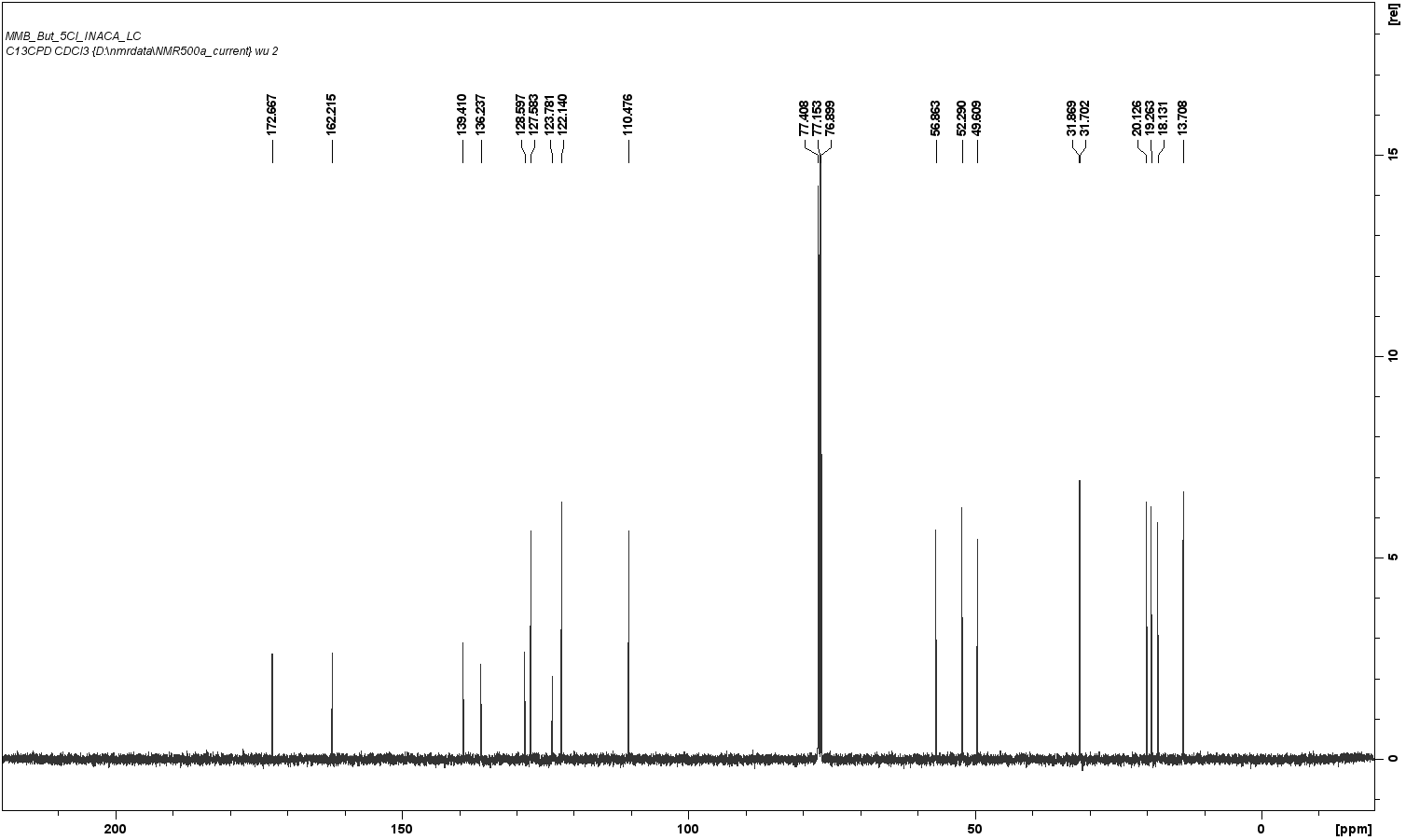
**Figure S2.6.** Chemical structure, ^1^H-NMR (500 MHz) spectrum, and ^13^C-NMR (126 MHz) spectrum for MMB-5’Cl-BUTINACA.


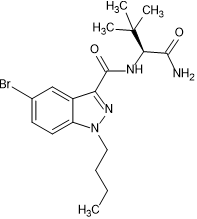

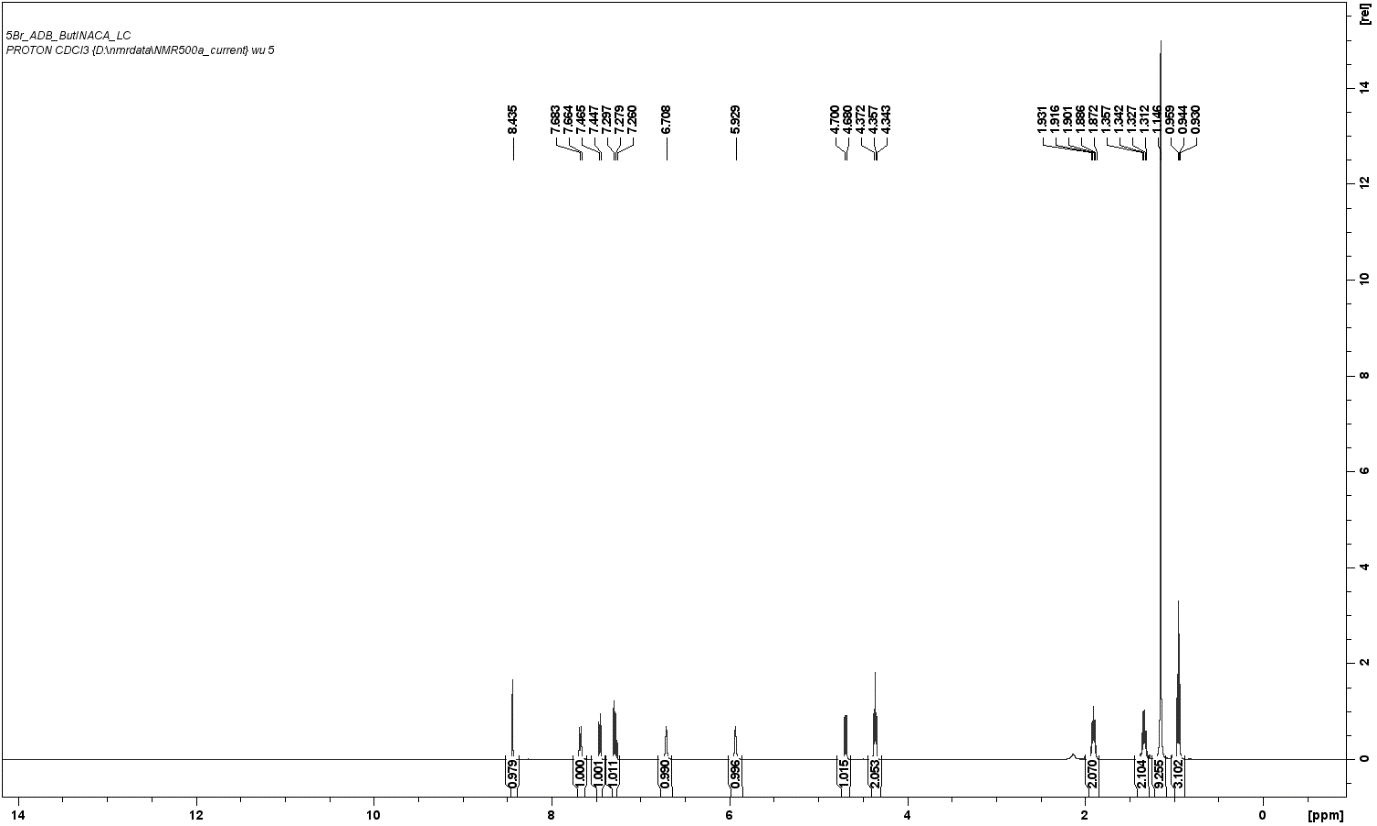


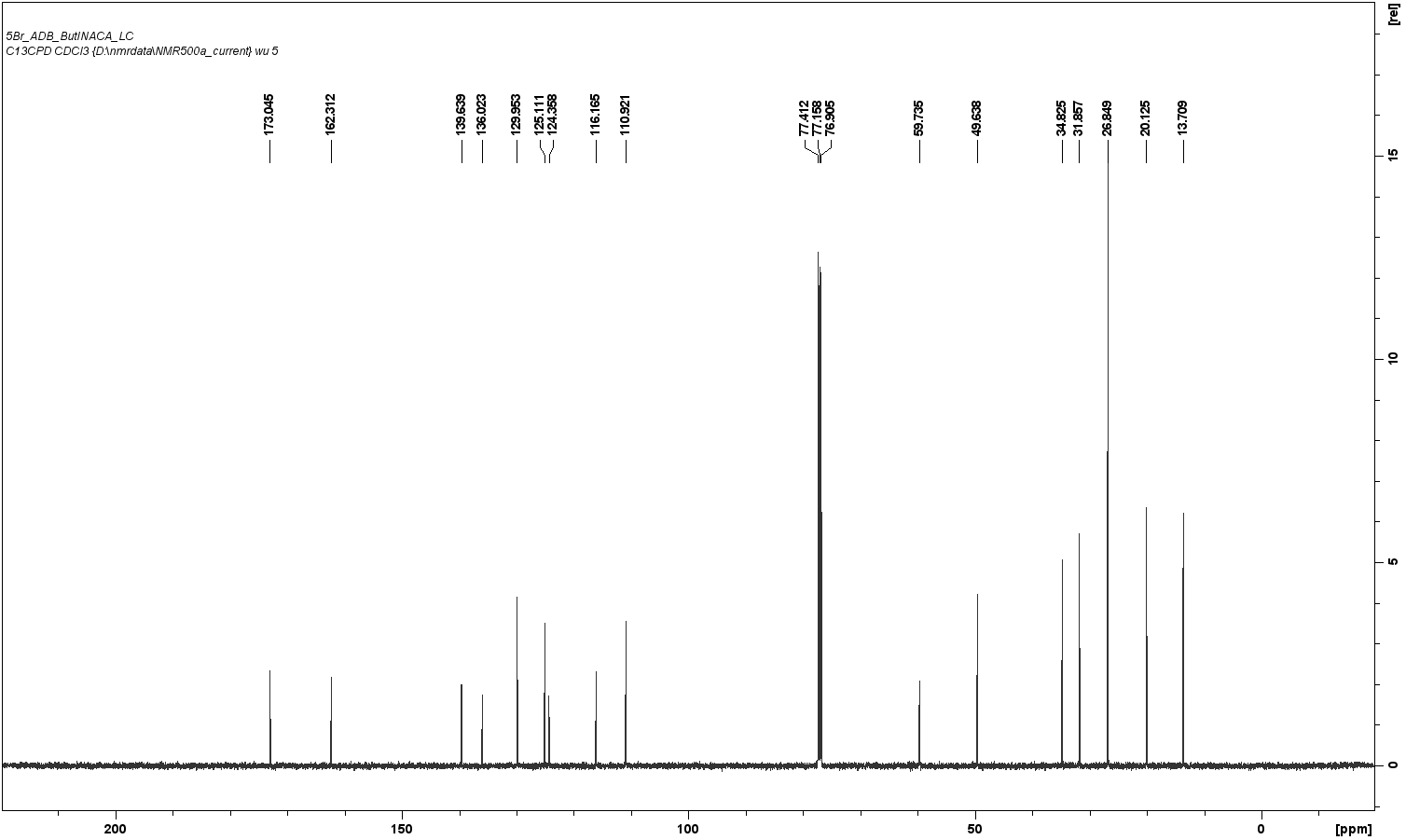
**Figure S2.7.** Chemical structure, ^1^H-NMR (500 MHz) spectrum, and ^13^C-NMR (126 MHz) spectrum for ADB-5’Br-BUTINACA.


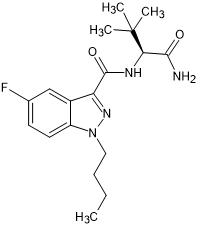

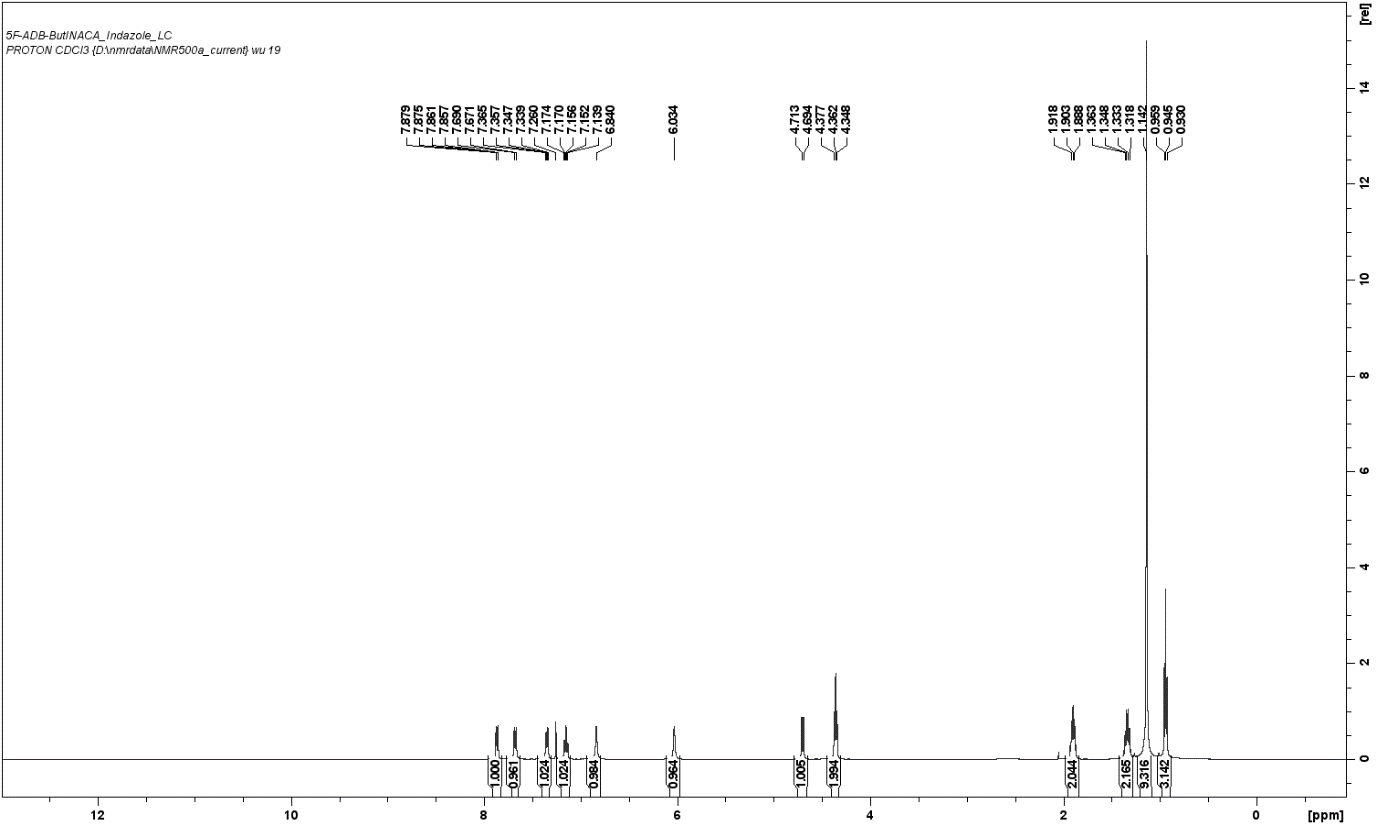


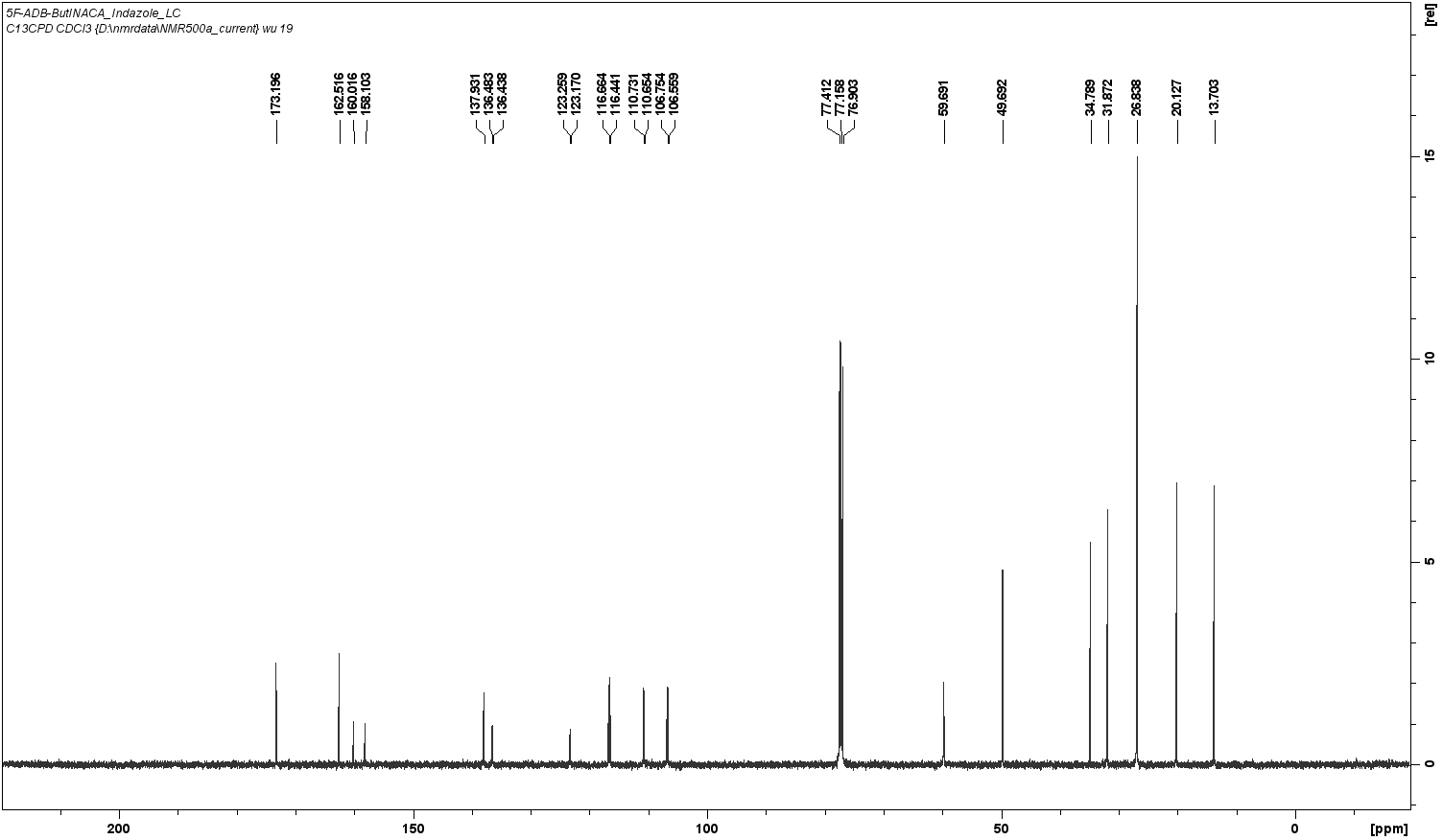
**Figure S2.8.** Chemical structure, ^1^H-NMR (500 MHz) spectrum, and ^13^C-NMR (126 MHz) spectrum for ADB-5’F-BUTINACA.


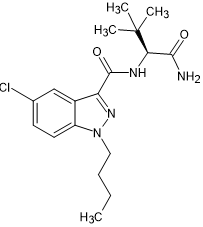

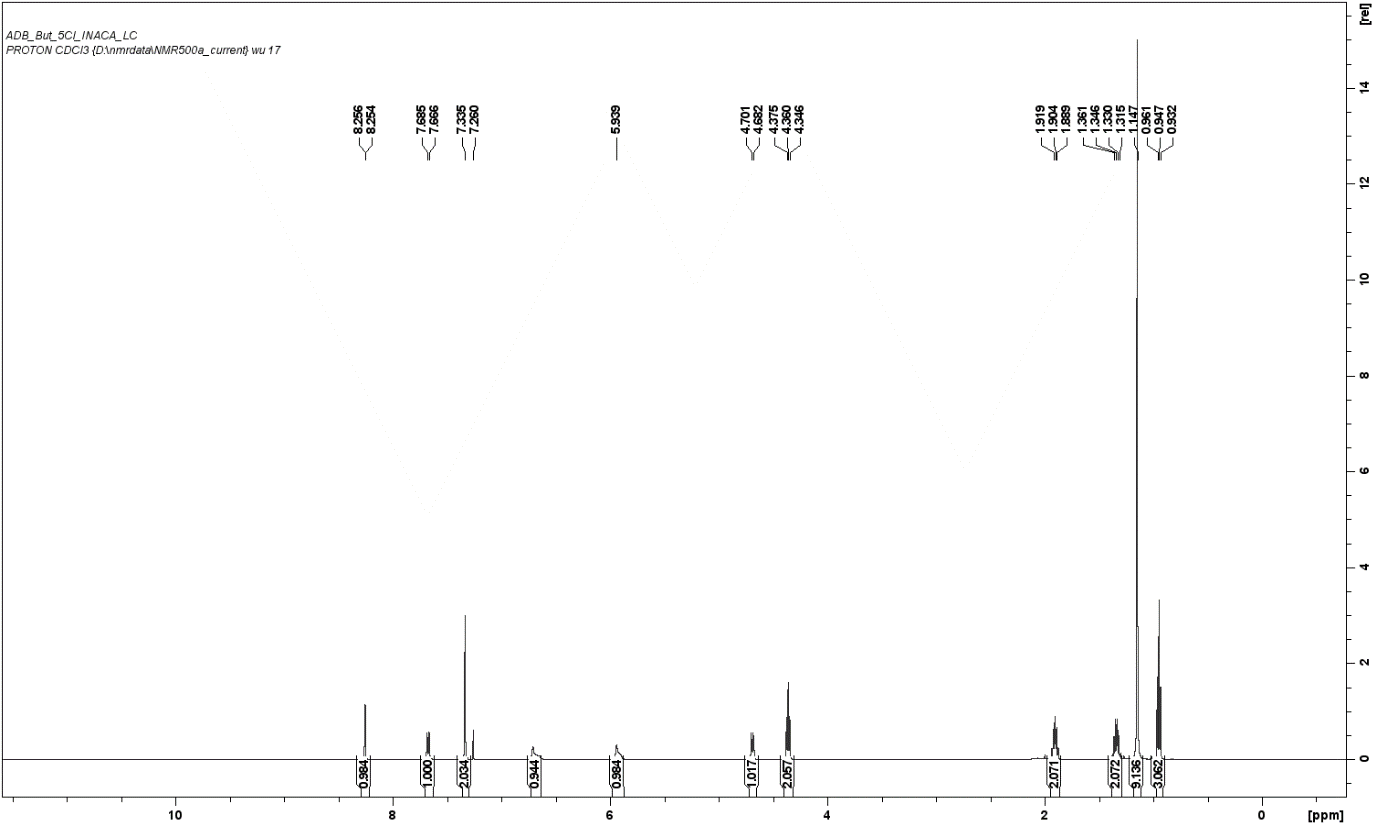


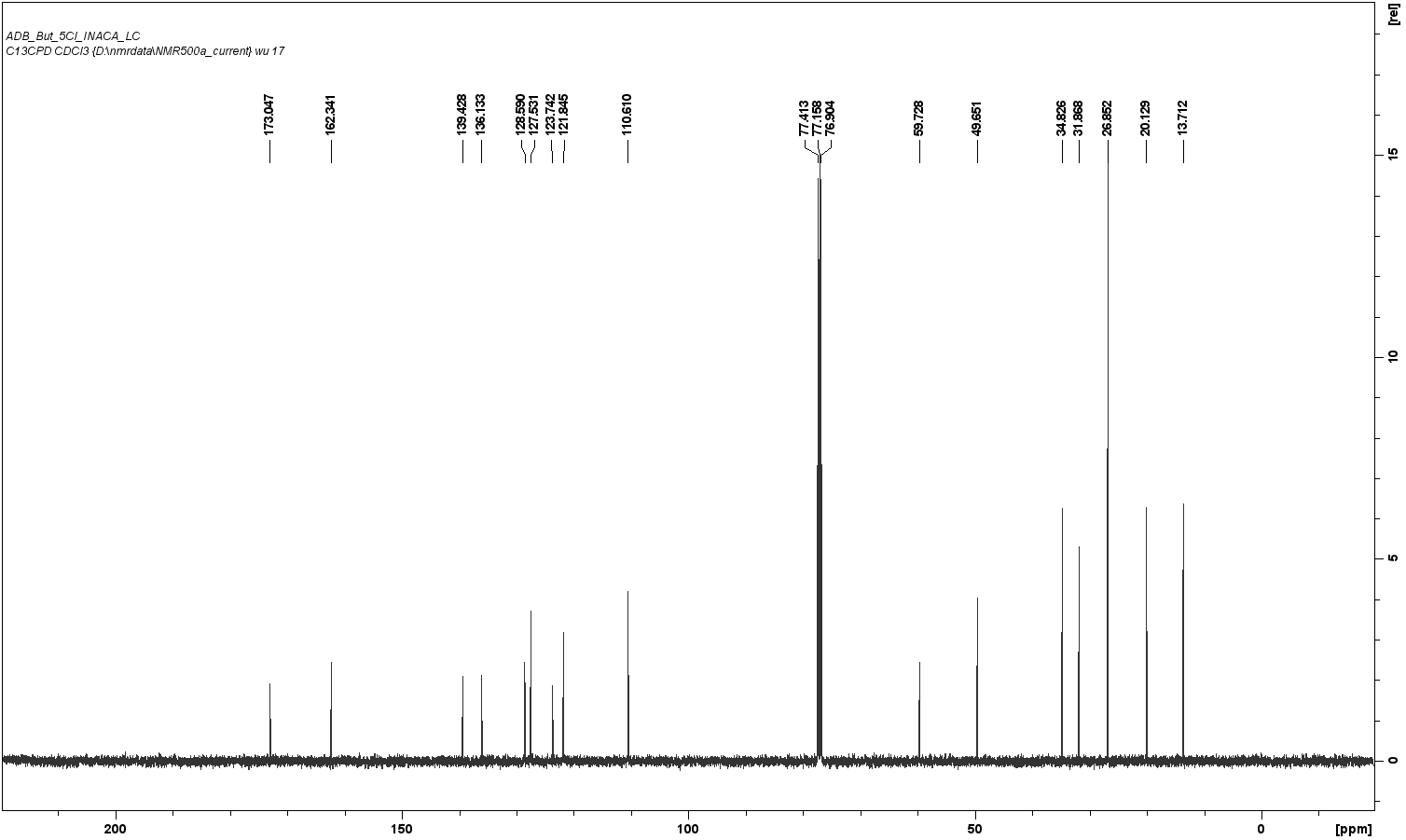
**Figure S2.9.** Chemical structure, ^1^H-NMR (500 MHz) spectrum, and ^13^C-NMR (126 MHz) spectrum for ADB-5’Cl-BUTINACA.


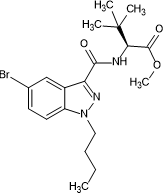

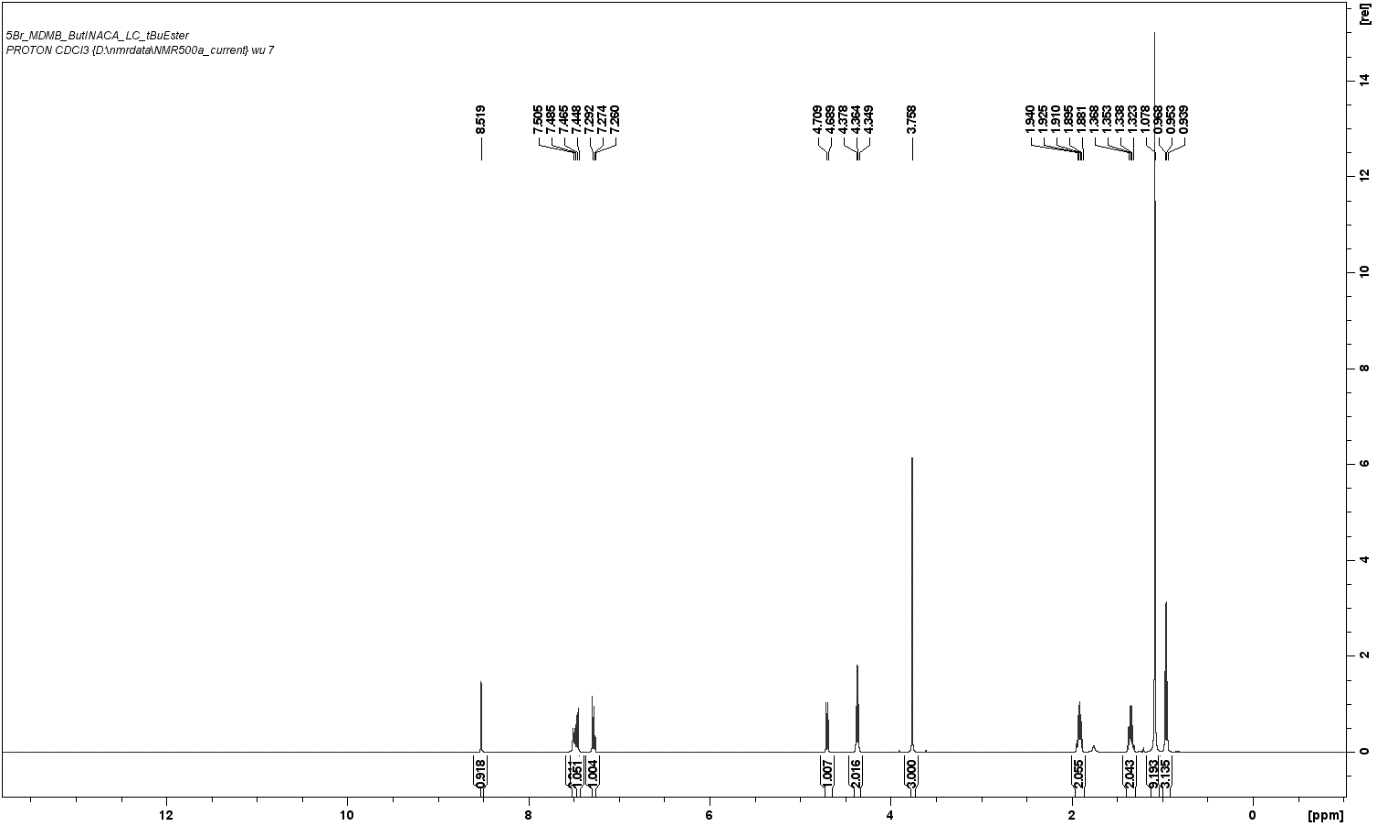


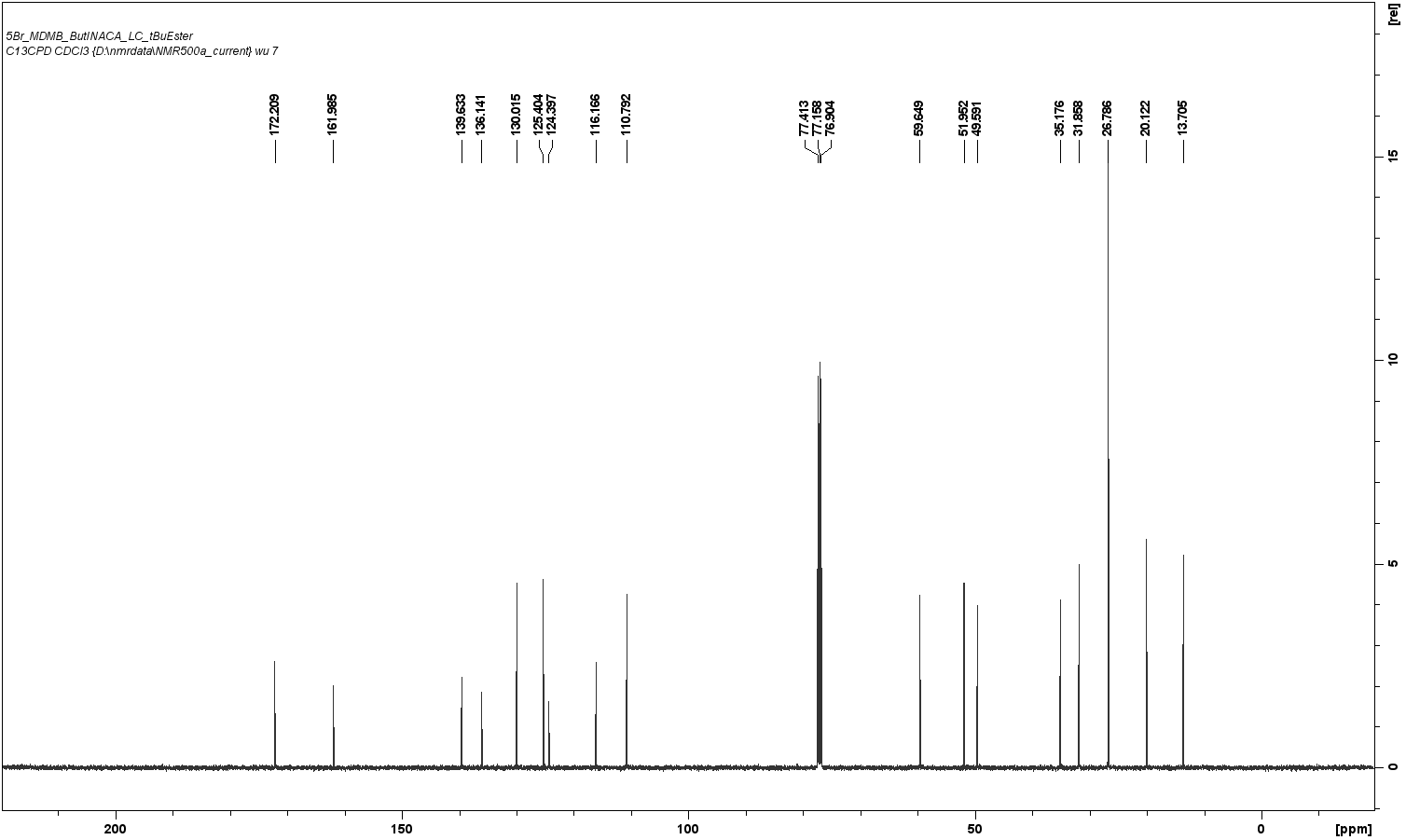
**Figure S2.10.** Chemical structure, ^1^H-NMR (500 MHz) spectrum, and ^13^C-NMR (126 MHz) spectrum for MDMB-5’Br-BUTINACA.


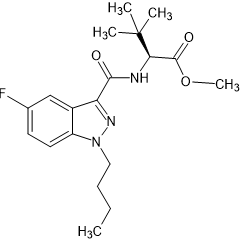

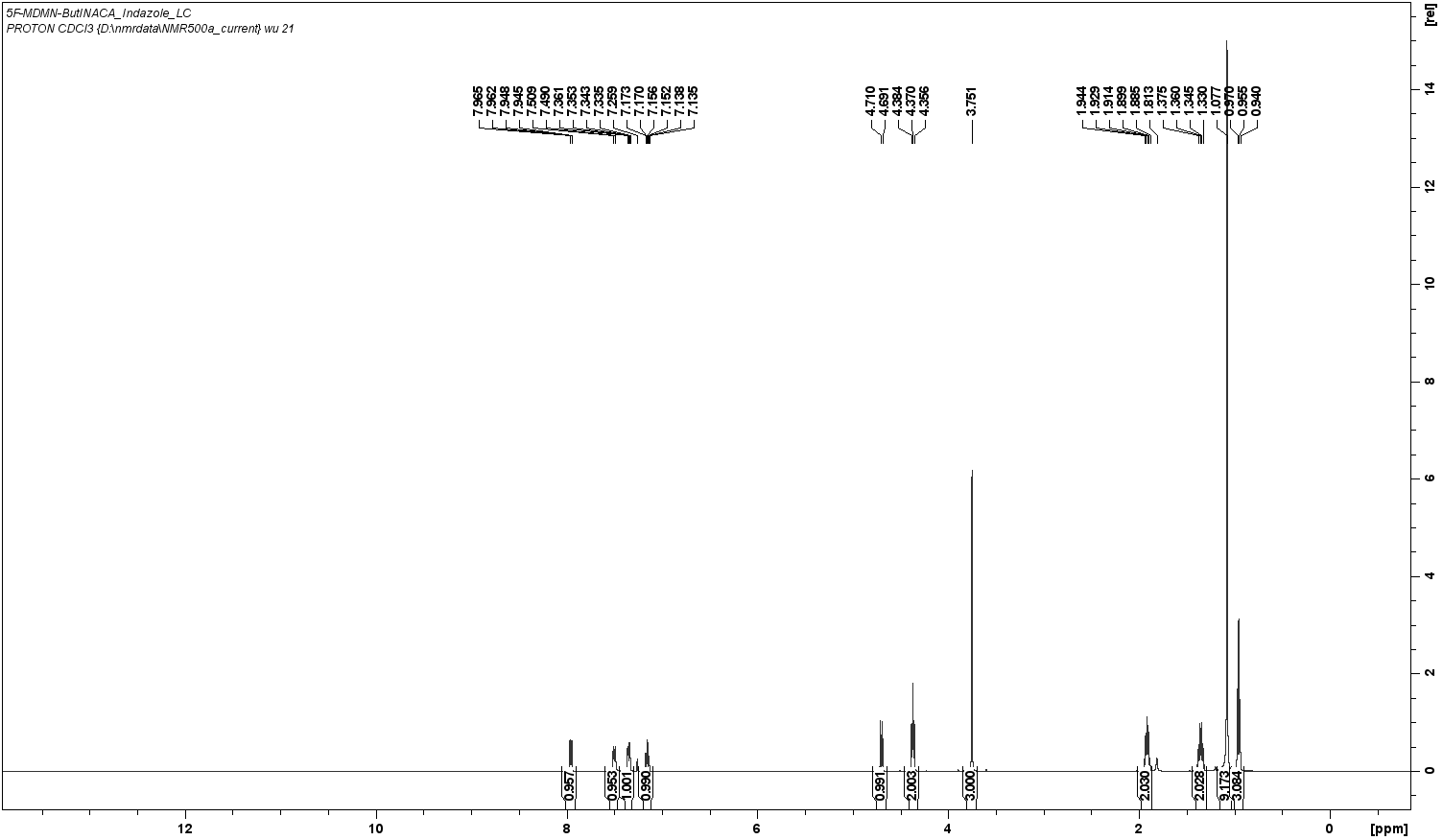


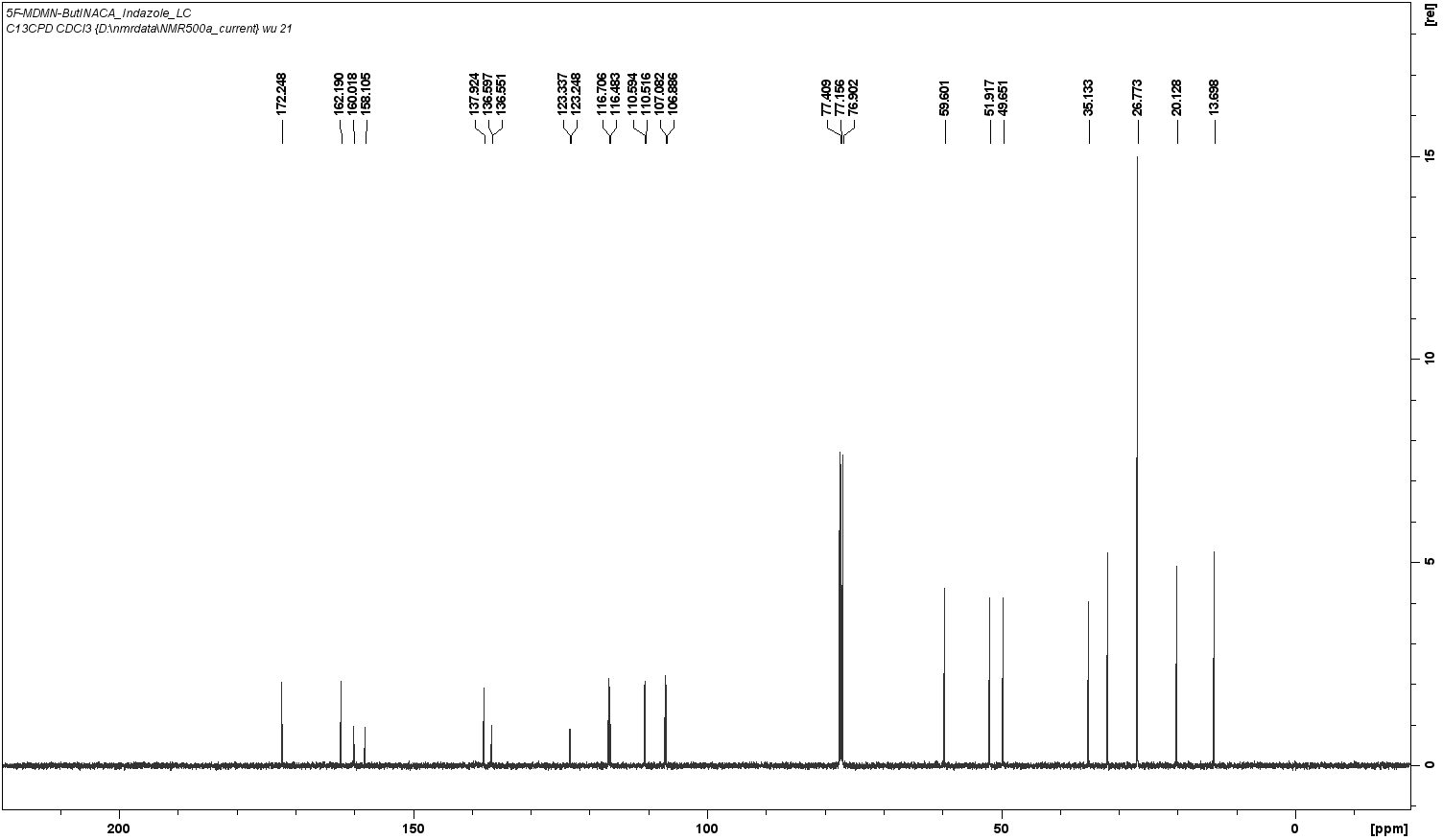
**Figure S2.11.** Chemical structure, ^1^H-NMR (500 MHz) spectrum, and ^13^C-NMR (126 MHz) spectrum for MDMB-5’F-BUTINACA.


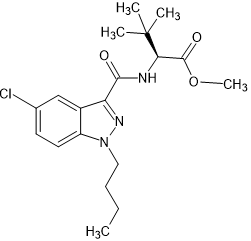

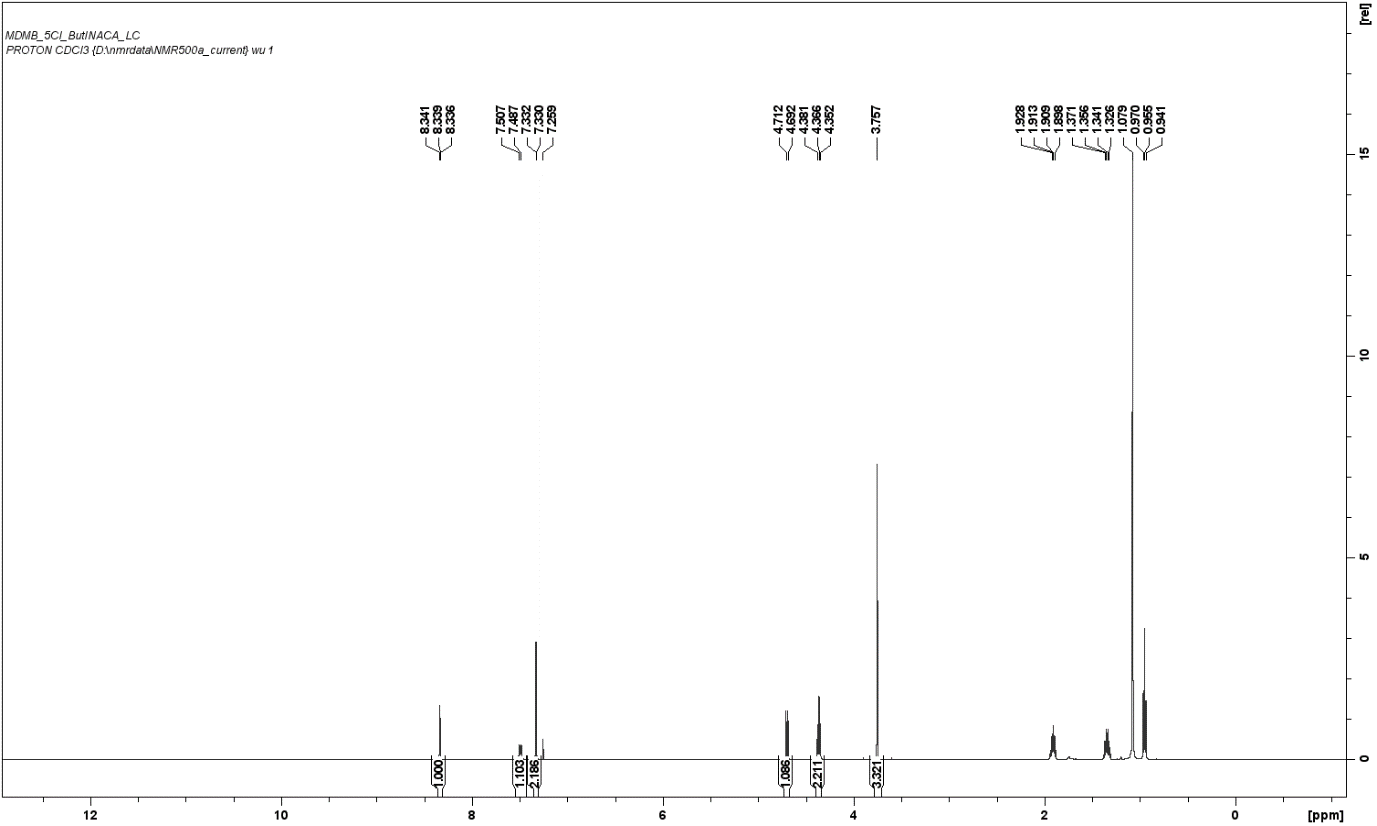


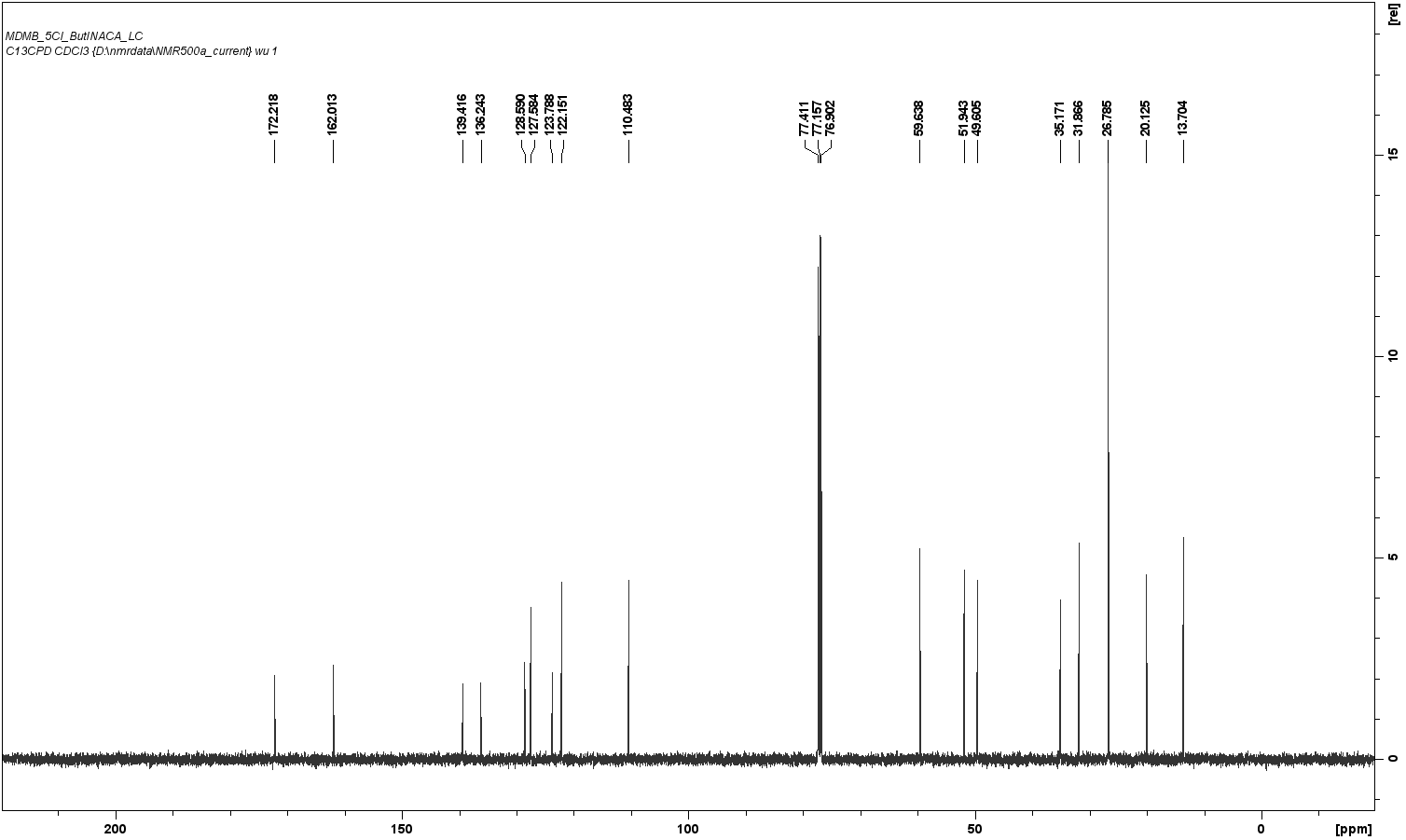
**Figure S2.12.** Chemical structure, ^1^H-NMR (500 MHz) spectrum, and ^13^C-NMR (126 MHz) spectrum for MDMB-5’Cl-BUTINACA.


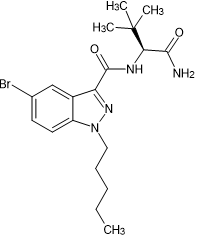

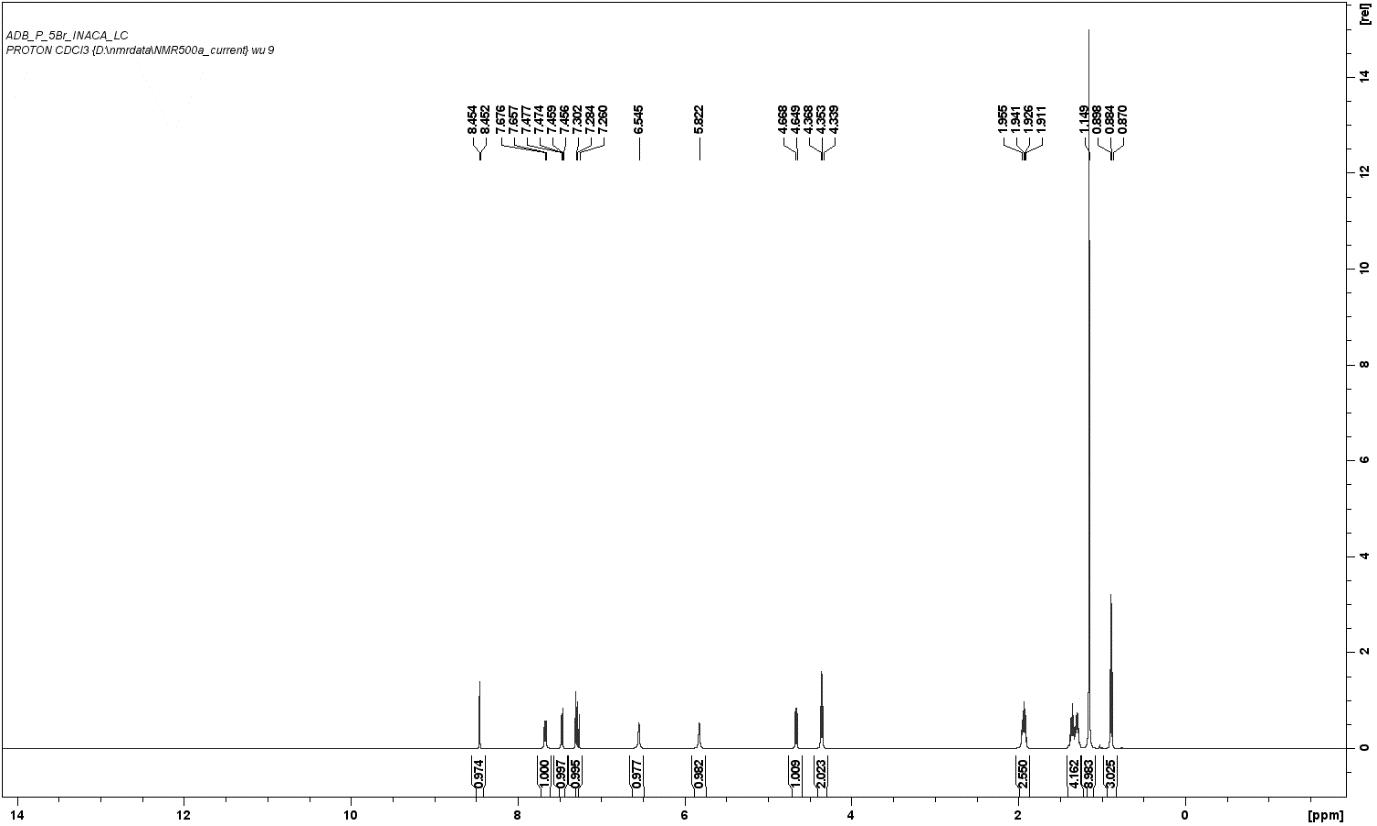


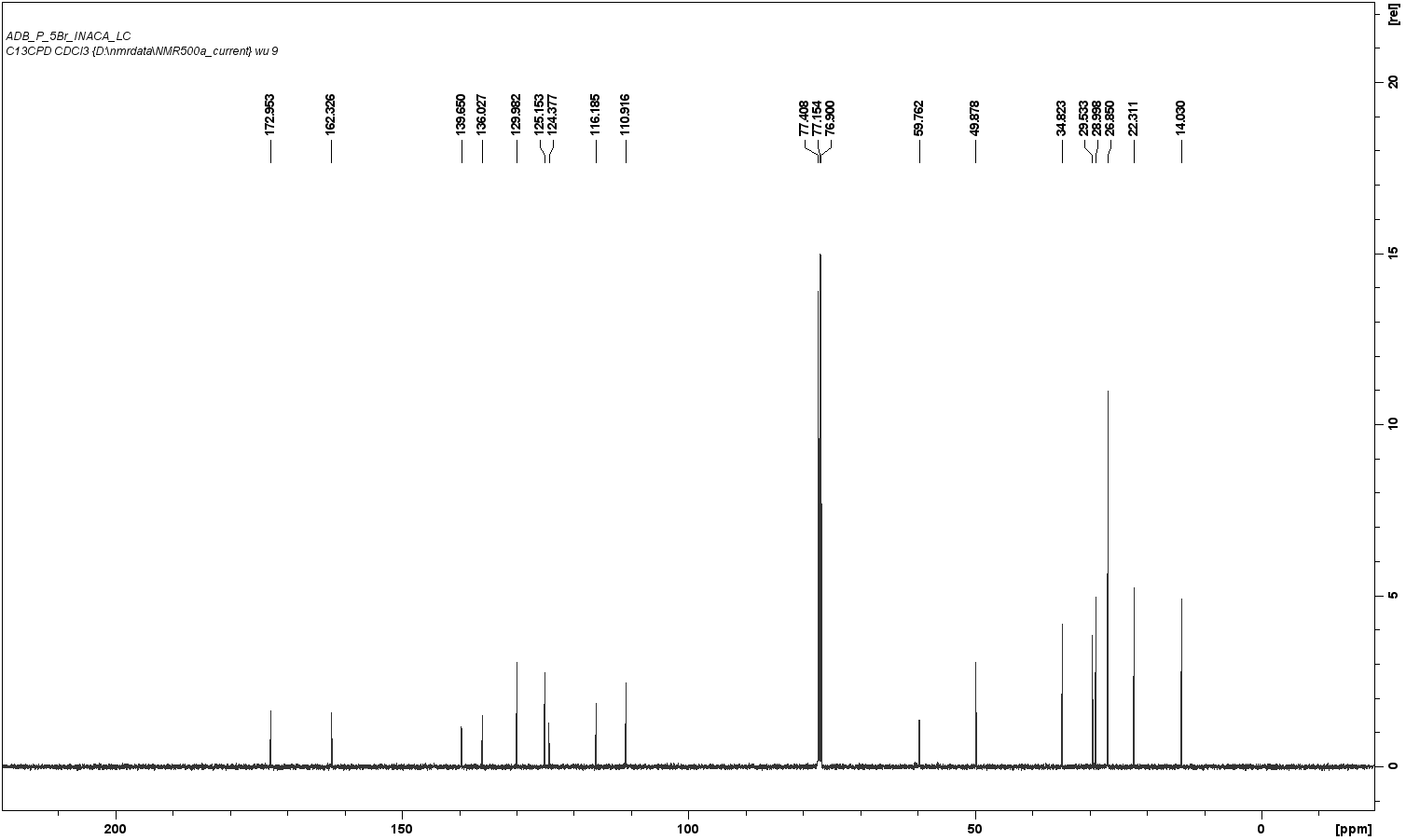
**Figure S2.13.** Chemical structure, ^1^H-NMR (500 MHz) spectrum, and ^13^C-NMR (126 MHz) spectrum for ADB-5’Br-PINACA.


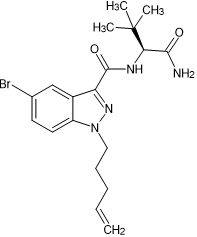

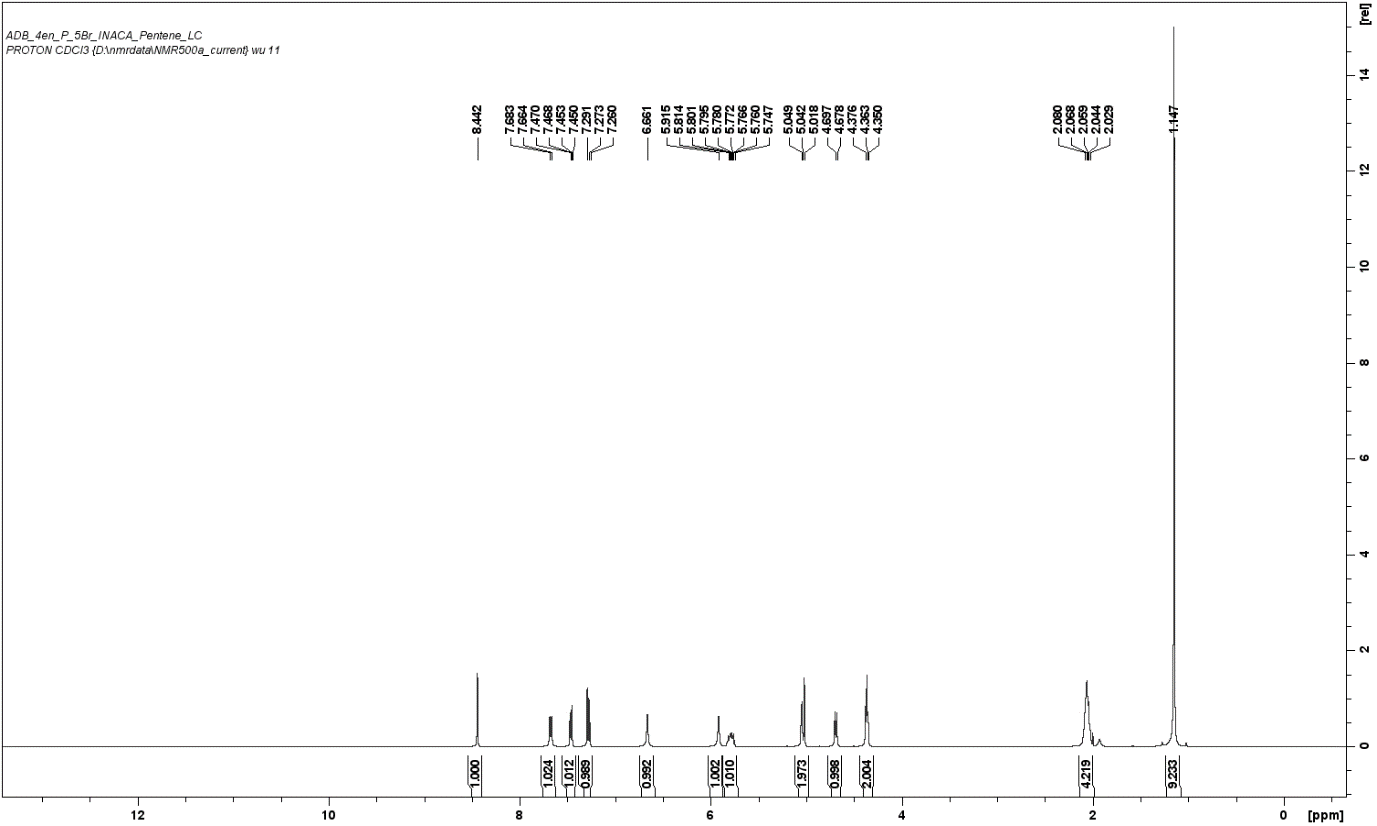


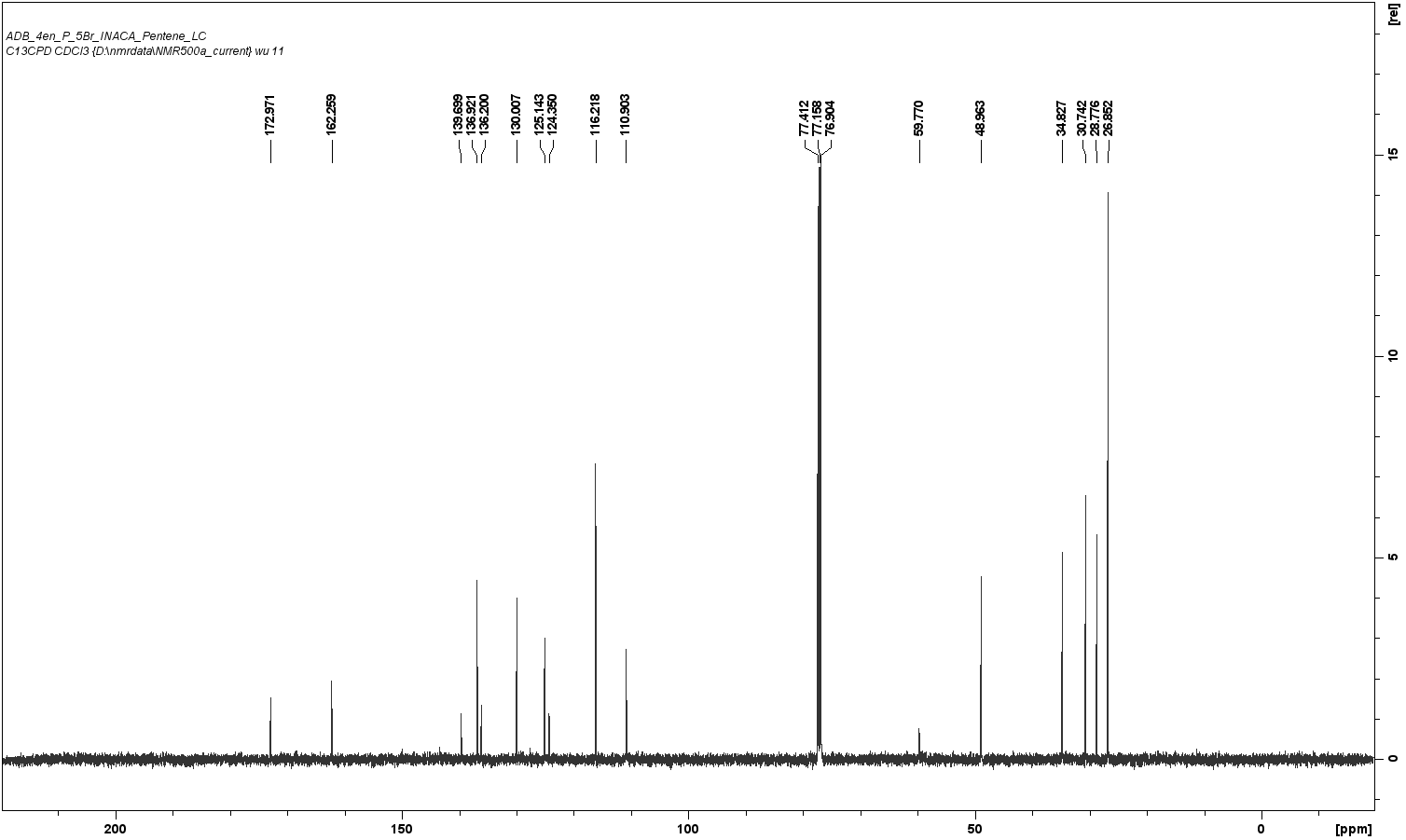
**Figure S2.14.** Chemical structure, ^1^H-NMR (500 MHz) spectrum, and ^13^C-NMR (126 MHz) spectrum for ADB-4en-5’Br-PINACA.


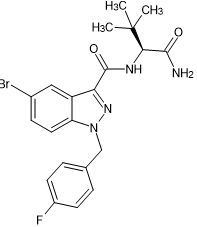

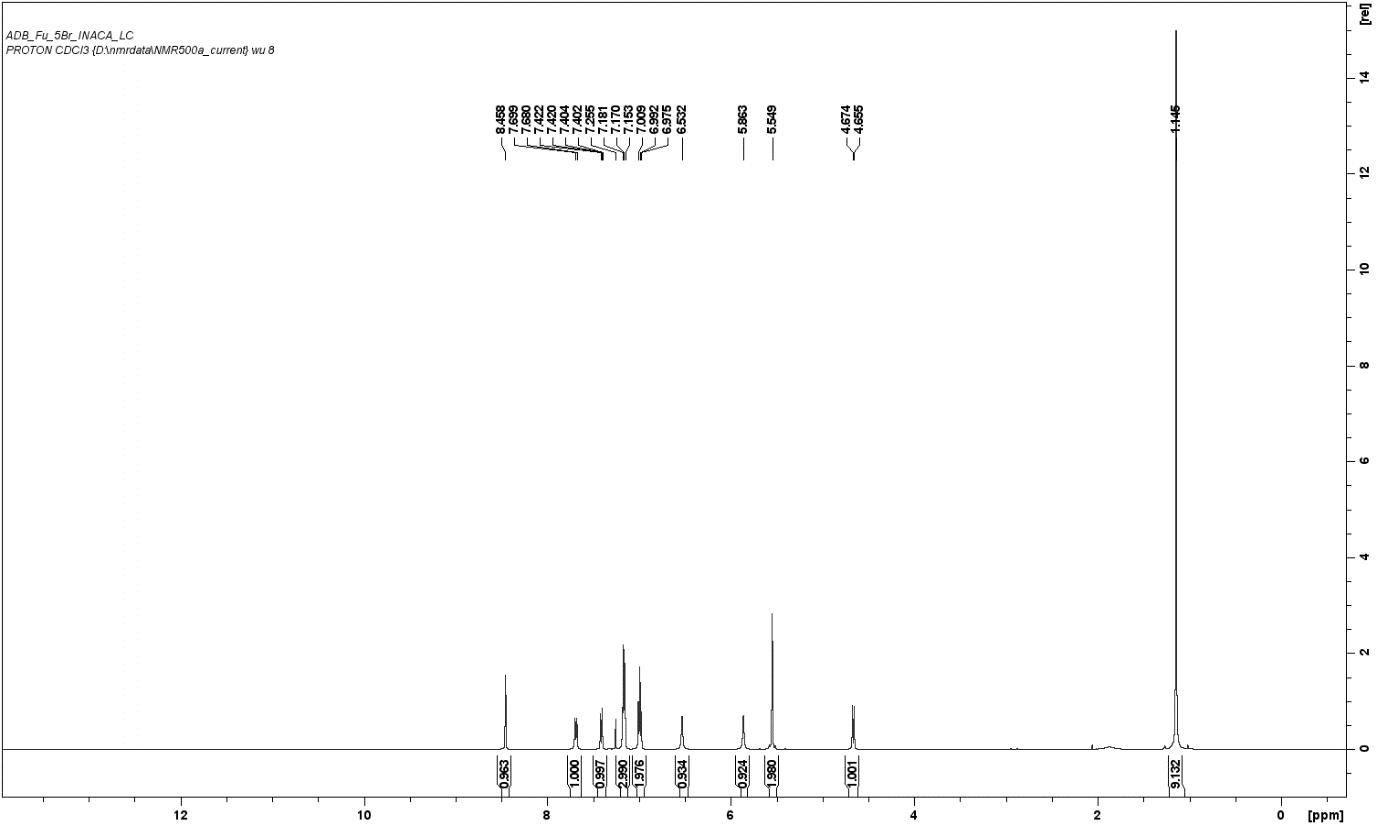


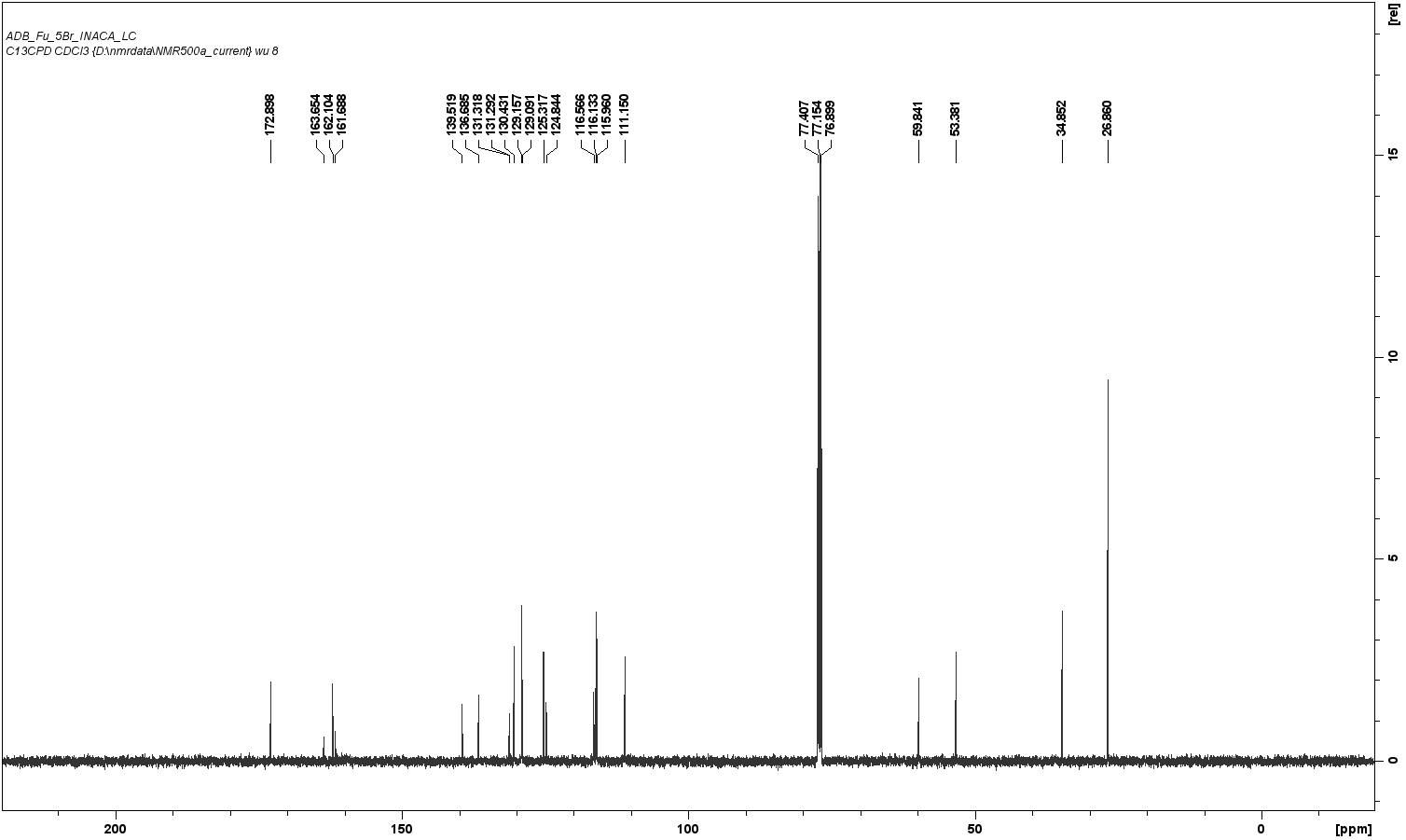
**Figure S2.15.** Chemical structure, ^1^H-NMR (500 MHz) spectrum, and ^13^C-NMR (126 MHz) spectrum for ADB-5’Br-FUBINACA.


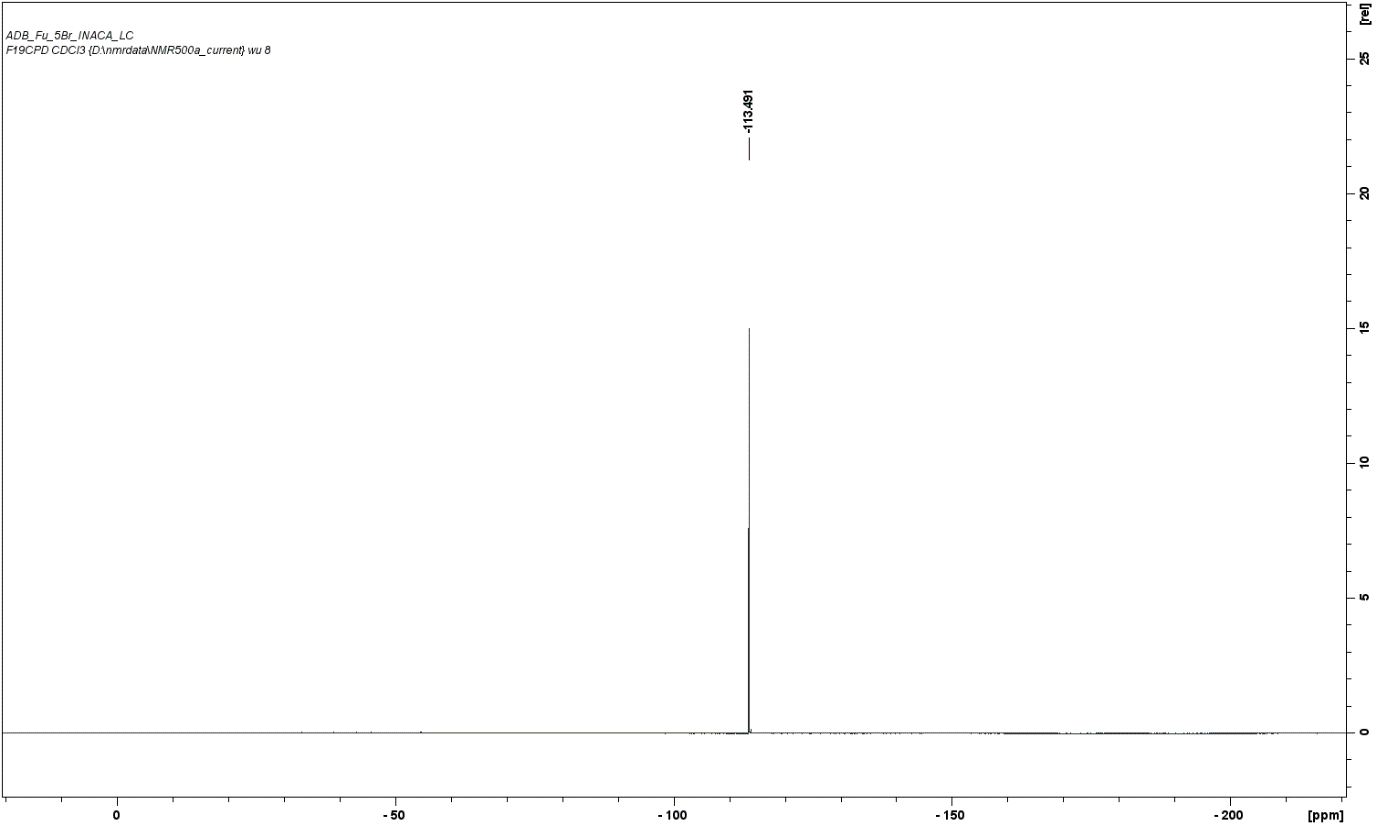
**Figure S2.16.** ^19^F-NMR spectrum for ADB-5’Br-FUBINACA.


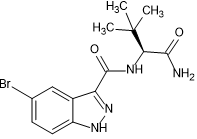

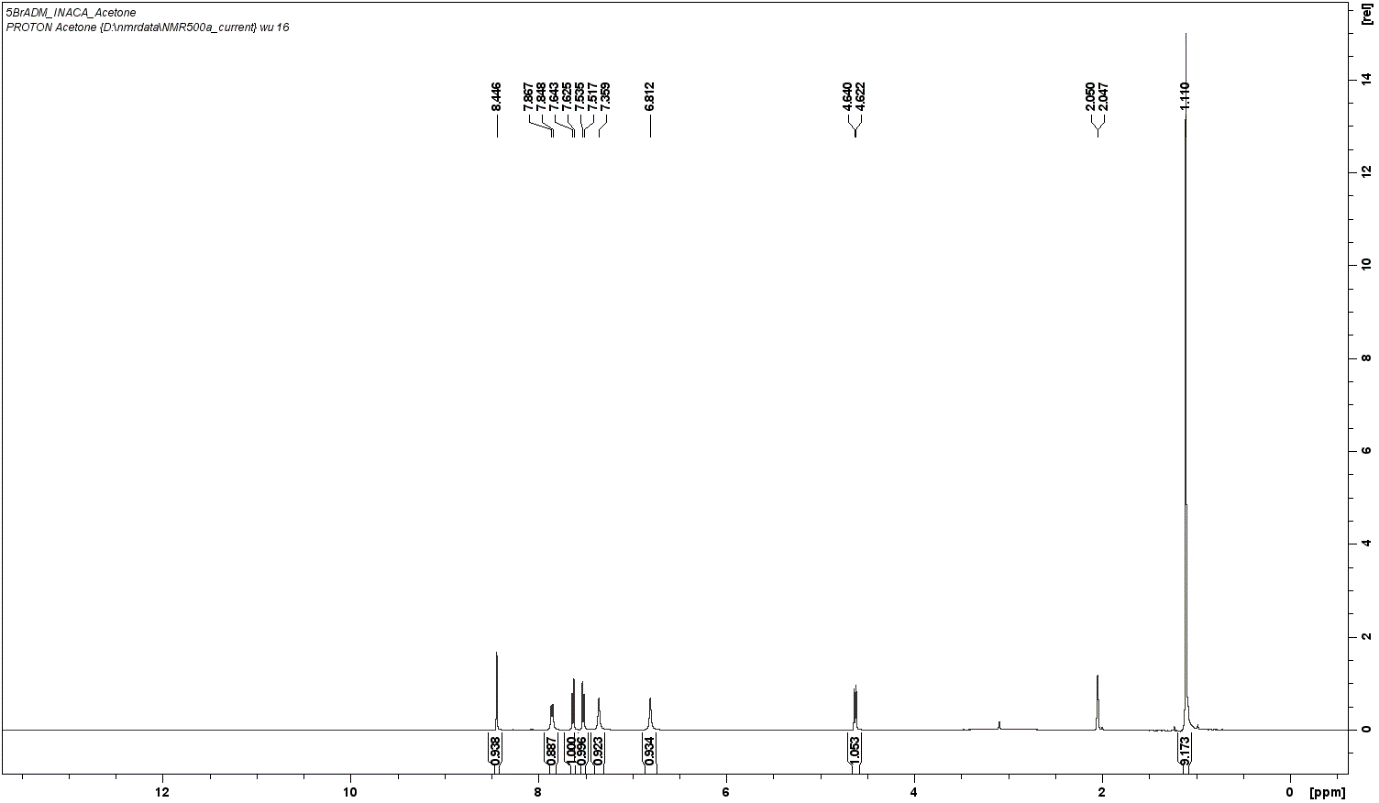


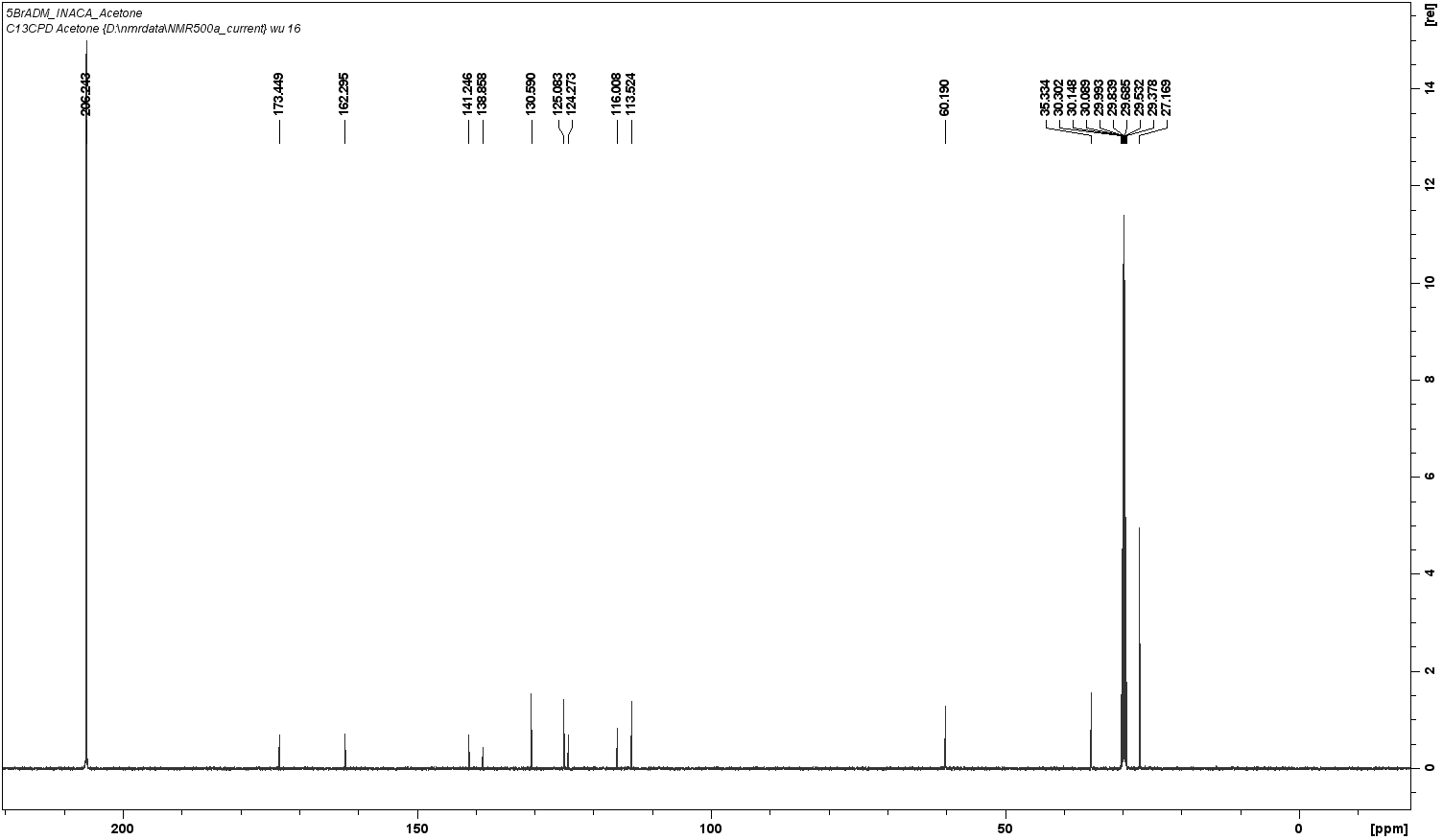
**Figure S2.17.** Chemical structure, ^1^H-NMR (500 MHz) spectrum, and ^13^C-NMR (126 MHz) spectrum for ADB-5’Br-INACA.

**Section 3: ANOVA results**

**Table S3.1.** Comparison of efficacy and potency between substitutions on the indazole core. P values are from Brown-Forsythe and Welch ANOVA tests with JWH-018 as a reference.

|  | **Comparisons** | | **Efficacy (E_max_)** | | | **Potency (EC_50_)** | | |
| --- | --- | --- | --- | --- | --- | --- | --- | --- |
|  | **Compound 1** | **Compound 2** | **Mean Diff** | **P value** | **Significant?** | **Mean Diff** | **P value** | **Significant?** |
| **Br vs. F** | AB-5’Br-BUTINACA | AB-5’F-BUTINACA | 7.90 | 0.0542 | No | 0.38 | 0.0092 | Yes |
|  | MMB-5’Br-BUTINACA | MMB-5’F-BUTINACA | -1.32 | 0.9558 | No | 0.99 | 0.0025 | Yes |
|  | ADB-5’Br-BUTINACA | ADB-5’F-BUTINACA | -3.19 | 0.7182 | No | 0.10 | 0.7016 | No |
|  | MDMB-5’Br-BUTINACA | MDMB-5’F-BUTINACA | 2.52 | 0.9575 | No | 0.87 | 0.0015 | Yes |
| **Br vs. Cl** | AB-5’Br-BUTINACA | AB-5’Cl-BUTINACA | 2.92 | 0.6150 | No | -0.11 | 0.3829 | No |
|  | MMB-5’Br-BUTINACA | MMB-5’Cl-BUTINACA | -3.81 | 0.6309 | No | 0.25 | 0.1336 | No |
|  | ADB-5’Br-BUTINACA | ADB-5’Cl-BUTINACA | -2.84 | 0.7007 | No | -0.22 | 0.0853 | No |
|  | MDMB-5’Br-BUTINACA | MDMB-5’Cl-BUTINACA | 3.79 | 0.6864 | No | 0.60 | 0.0122 | Yes |
| **F vs. Cl** | AB-5’F-BUTINACA | AB-5’Cl-BUTINACA | -4.99 | 0.2065 | No | -0.49 | 0.0037 | Yes |
|  | MMB-5’F-BUTINACA | MMB-5’Cl-BUTINACA | -2.49 | 0.5978 | No | -0.75 | 0.0003 | Yes |
|  | ADB-5’F-BUTINACA | ADB-5’Cl-BUTINACA | 0.36 | >0.9999 | No | -0.32 | 0.0631 | No |
|  | MDMB-5’F-BUTINACA | MDMB-5’Cl-BUTINACA | 1.27 | 0.9983 | No | -0.26 | 0.0594 | No |
| **Br vs. H** | ADB-5’Br-BUTINACA | ADB-BUTINACA | -15.66 | 0.0056 | Yes | -0.08 | 0.6638 | No |
|  | MDMB-5’Br-BUTINACA | MDMB-BUTINACA | -7.53 | 0.2634 | No | 0.68 | 0.0053 | Yes |
|  | MDMB-4en-5’Br-PINACA | MDMB-4en-PINACA | 11.19 | 0.0353 | Yes | 0.40 | 0.0109 | Yes |
|  | MDMB-5’Br-INACA | MDMB-INACA | -2.31 | 0.9919 | No | -0.22 | 0.0556 | No |
| **F vs. H** | ADB-5’F-BUTINACA | ADB-BUTINACA | -12.47 | 0.0358 | Yes | -0.17 | 0.3268 | No |
|  | MDMB-5’F-BUTINACA | MDMB-BUTINACA | -10.05 | 0.0758 | No | -0.19 | 0.2996 | No |
| **Cl vs. H** | ADB-5’Cl-BUTINACA | ADB-BUTINACA | -12.82 | 0.0217 | Yes | 0.15 | 0.2990 | No |
|  | MDMB-5’Cl-BUTINACA | MDMB-BUTINACA | -11.32 | 0.0723 | No | 0.07 | 0.9263 | No |
|  | MDMB-5’Me-INACA | MDMB-INACA | 1.15 | >0.9999 | No | 0.43 | 0.0675 | No |
|  | MDMB-5’Me-INACA | MDMB-5’Br-INACA | 3.46 | 0.9908 | No | 0.21 | 0.3643 | No |

**Table S3.2.** Comparison of efficacy and potency between different head moieties. P values are from Brown-Forsythe and Welch ANOVA tests with JWH-018 as a reference.

|  | **Comparisons** | | **Efficacy (E_max_)** | | | **Potency (EC_50_)** | | |
| --- | --- | --- | --- | --- | --- | --- | --- | --- |
|  | **Compound 1** | **Compound 2** | **Mean Diff** | **P value** | **Significant?** | **Mean Diff** | **P value** | **Significant?** |
| **AB**  **vs.**  **MMB** | AB-5’Br-BUTINACA | MMB-5’Br-BUTINACA | 9.47 | 0.2199 | No | -0.76 | 0.0112 | Yes |
|  | AB-5’F-BUTINACA | MMB-5’F-BUTINACA | 0.24 | >0.9999 | No | -0.15 | 0.1612 | No |
|  | AB-5’Cl-BUTINACA | MMB-5’Cl-BUTINACA | 2.74 | 0.8336 | No | -0.41 | 0.0130 | Yes |
| **AB**  **vs.**  **ADB** | AB-5’Br-BUTINACA | ADB-5’Br-BUTINACA | 5.08 | 0.2732 | No | 0.39 | 0.0113 | Yes |
|  | AB-5’F-BUTINACA | ADB-5’F-BUTINACA | -6.01 | 0.3008 | No | 0.11 | 0.6242 | No |
|  | AB-5’Cl-BUTINACA | ADB-5’Cl-BUTINACA | -0.67 | 0.9998 | No | 0.28 | 0.0621 | Yes |
| **AB**  **vs.**  **MDMB** | AB-5’Br-BUTINACA | MDMB-5’Br-BUTINACA | 1.26 | 0.9916 | No | 0.02 | 0.9993 | No |
|  | AB-5’F-BUTINACA | MDMB-5’F-BUTINACA | -4.12 | 0.2965 | No | 0.51 | 0.0037 | Yes |
|  | AB-5’Cl-BUTINACA | MDMB-5’Cl-BUTINACA | 2.14 | 0.8537 | No | 0.74 | 0.0038 | Yes |
| **MMB**  **vs.**  **ADB** | MMB-5’Br-BUTINACA | ADB-5’Br-BUTINACA | -4.38 | 0.6673 | No | 1.16 | 0.0027 | Yes |
|  | MMB-5’F-BUTINACA | ADB-5’F-BUTINACA | -6.25 | 0.6540 | No | 0.26 | 0.1151 | Yes |
|  | MMB-5’Cl-BUTINACA | ADB-5’Cl-BUTINACA | -3.40 | 0.6823 | No | 0.69 | 0.0018 | Yes |
| **MMB**  **vs.**  **MDMB** | MMB-5’Br-BUTINACA | MDMB-5’Br-BUTINACA | -3.20 | 0.2897 | No | 0.79 | 0.0102 | Yes |
|  | MMB-5’F-BUTINACA | MDMB-5’F-BUTINACA | -4.36 | 0.2094 | No | 0.66 | 0.0010 | Yes |
|  | MMB-5’Cl-BUTINACA | MDMB-5’Cl-BUTINACA | -0.60 | 0.9994 | No | 1.15 | <0.0001 | Yes |
| **ADB**  **vs.**  **MDMB** | ADB-5’Br-BUTINACA | MDMB-5’Br-BUTINACA | -3.82 | 0.4620 | No | -0.37 | 0.0134 | Yes |
|  | ADB-5’F-BUTINACA | MDMB-5’F-BUTINACA | 1.89 | 0.8004 | No | 0.40 | 0.0454 | Yes |
|  | ADB-5’Cl-BUTINACA | MDMB-5’Cl-BUTINACA | 2.80 | 0.6783 | No | 0.46 | 0.0159 | Yes |
|  | ADB-5’Br-4en-PINACA | MDMB-5’Br-4en-PINACA | -8.94 | 0.0881 | No | -7.85 | <0.0001 | Yes |

**Table S3.3.** Comparison of efficacy and potency between different tail moieties. P values are from Brown-Forsythe and Welch ANOVA tests with JWH-018 as a reference.

| **Comparisons** | | **Efficacy (E_max_)** | | | **Potency (EC_50_)** | | |
| --- | --- | --- | --- | --- | --- | --- | --- |
| **Compound 1** | **Compound 2** | **Mean Diff** | **P value** | **Significant?** | **Mean Diff** | **P value** | **Significant?** |
| ADB-5’Br-INACA | ADB-5’Br-BUTINACA | 1.04 | >0.9999 | No | 2.10 | <0.0001 | Yes |
| ADB-5’Br-INACA | ADB-5’Br-FUBINACA | -1.25 | >0.9999 | No | 1.74 | <0.0001 | Yes |
| ADB-5’Br-INACA | ADB-5’Br-PINACA | 2.88 | 0.8707 | No | 2.06 | <0.0001 | Yes |
| ADB-5’Br-INACA | ADB-4en-5’Br-PINACA | -0.21 | >0.9999 | No | 2.21 | <0.0001 | Yes |
| ADB-5’Br-BUTINACA | ADB-5’Br-FUBINACA | -2.30 | 0.9578 | No | -0.37 | 0.0271 | Yes |
| ADB-5’Br-BUTINACA | ADB-5’Br-PINACA | 1.84 | 0.9387 | No | -0.04 | 0.9906 | No |
| ADB-5’Br-BUTINACA | ADB-4en-5’Br-PINACA | -1.26 | 0.9975 | No | 0.10 | 0.5606 | No |
| ADB-5’Br-FUBINACA | ADB-5’Br-PINACA | 4.13 | 0.5768 | No | 0.33 | 0.0637 | No |
| ADB-5’Br-FUBINACA | ADB-4en-5’Br-PINACA | 1.04 | >0.9999 | No | 0.47 | 0.0112 | Yes |
| ADB-5’Br-PINACA | ADB-4en-5’Br-PINACA | -3.10 | 0.6127 | No | 0.14 | 0.2445 | No |
| MDMB-5’Br-BUTINACA | MDMB-4en-5’Br-PINACA | -6.38 | 0.2729 | No | -0.02 | 0.9990 | No |
